# Photodissociation of Sodium Iodide Clusters Doped with Small Hydrocarbons

**DOI:** 10.1002/chem.201803017

**Published:** 2018-07-27

**Authors:** Nina K. Bersenkowitsch, Milan Ončák, Jakob Heller, Christian van der Linde, Martin K. Beyer

**Affiliations:** ^1^ Institut für Ionenphysik und Angewandte Physik Universität Innsbruck Technikerstraße 25 6020 Innsbruck Austria

**Keywords:** ab initio calculations, marine aerosols, mass spectrometry, photodissociation, spectroscopy

## Abstract

Marine aerosols consist of a variety of compounds and play an important role in many atmospheric processes. In the present study, sodium iodide clusters with their simple isotope pattern serve as model systems for laboratory studies to investigate the role of iodide in the photochemical processing of sea‐salt aerosols. Salt clusters doped with camphor, formate and pyruvate are studied in a Fourier transform ion cyclotron resonance mass spectrometer (FT‐ICR MS) coupled to a tunable laser system in both UV and IR range. The analysis is supported by ab initio calculations of absorption spectra and energetics of dissociative channels. We provide quantitative analysis of IRMPD measurements by reconstructing one‐photon spectra and comparing them with the calculated ones. While neutral camphor is adsorbed on the cluster surface, the formate and pyruvate ions replace an iodide ion. The photodissociation spectra revealed several wavelength‐specific fragmentation pathways, including the carbon dioxide radical anion formed by photolysis of pyruvate. Camphor and pyruvate doped clusters absorb in the spectral region above 290 nm, which is relevant for tropospheric photochemistry, leading to internal conversion followed by intramolecular vibrational redistribution, which leads to decomposition of the cluster. Potential photodissociation products of pyruvate in the actinic region may be formed with a cross section of <2×10^−20^ cm^2^, determined by the experimental noise level.

## Introduction

Aerosols play an important part in atmospheric chemistry,^1^, ^2^, ^3^ and have significant effect on the climate.^4^, ^5^ Examples are backscattering of solar radiation by clustered sea salt particles^6^ or photochemical processing of organic matter.^7^ Since the oceans cover more than 70 % of the Earth′s surface, one of the most important type of aerosols for the development of the climate on Earth are marine aerosols. Primary sea salt aerosols are produced by the mechanical disruption of ocean surface.^8^ Depending on the wind speed, sea salt aerosols in the range of 0.1–100 μm are obtained. Secondary aerosols contain non‐sea salt species like sulfate and organic compounds, formed by gas‐to‐particle conversion processes.^8^

Marine aerosols^9^ consist mainly of NaCl and of an enormous variety of organic compounds.^6^ Several studies reported significant concentrations of organic matter in aerosols.^10^, ^11^, ^12^, ^13^ In the sea spraying process, very tiny droplets containing water, salt and other organic and inorganic species are produced. After the evaporation of water, mainly salt clusters with hydrocarbons attached remain. Photochemical processing of organic matter then occurs on the surface of the aerosol.

Besides chloride, other halide ions like iodide are present in traces in sea‐salt aerosols, and may alter their chemistry and photochemistry. The investigation of alkali halide clusters in the gas phase was pioneered by Whetten and Martin and further followed by other authors.^14^, ^15^, ^16^, ^17^ Particularly well studied is the adsorption of ammonia^18^, ^19^ and water^20^, ^21^, ^22^, ^23^, ^24^, ^25^ to a variety of alkali halide species. Drewello and co‐workers studied the stability of salt clusters attached to crown ether decorated phthalocyanines.^26^ Sodium iodide clusters were subject of several gas phase studies. Ouyang et al. investigated the collision cross section with mobility measurements^27^ and Blades et al. studied the hydration energy of Na_2_I^+^.^28^ Ab initio calculations concerning the stability and structure were performed by Aguado et al. on singly charged^29^ and neutral^30^ sodium iodide clusters. Misaizu et al. investigated the structures of Na_*n*_I_*n*−1_^+^ with adsorbed methanol by photodissociation and density functional theory.^31^

We have recently studied the photochemistry of glyoxylate embedded in sodium chloride clusters as a laboratory model for the photochemical processing of organic matter in marine aerosols.^32^ In the present study, sodium iodide clusters are chosen as a model for sea salt aerosols to investigate a potentially active role of iodide interacting with hydrocarbons. In particular, NaI absorbs in the wavelength region of the laser system used here above 225 nm, which may lead to photochemical reactions of the organic guest with the electronically excited cluster. Photofragmentation is induced by irradiating positively charged sodium iodide clusters doped with hydrocarbons, and the resulting products are examined as a function of wavelength. In the infrared part of the spectrum, we used the infrared multiple photon dissociation technique (IRMPD) that has been already employed to study similar systems, for example methane on metal clusters.^33^, ^34^ The three hydrocarbons chosen for the present study are camphor, formic acid and pyruvic acid.

Camphor was observed at levels of around 10 pptv in the 1997 Southern California Ozone Study.^35^ The photolysis of camphor was investigated in solution by Agosta et al.^36^ where they found several photolytic products. Rate constants for the reaction of camphor with OH radicals, NO_3_ radicals and O_3_ were investigated by Reissell et al.^37^ They found that the dominant process for atmospheric loss of camphor is through reactions with the OH radical, and acetone was identified as reaction product.^37^

There is evidence that organic acids play an important role in the formation of cloud condensation nuclei.^38^ Formic acid and acetic acid are the dominant carboxylic acids in the troposphere.^39^ The photochemistry of formic acid was investigated in rare gas matrices^40^, ^41^ and dissociation channels of H_2_+CO_2_ and H_2_O+CO were identified. We recently showed that formic acid is oxidized by the carbonate radical anion CO_3_^.−^ to CO_2_ and H_2_O, and the detached electron is available for the formation of a new CO_3_^.−^ molecular ion.^42^

Pyruvic acid is atmospherically relevant since it is emitted in the atmosphere by several processes like biomass burning^43^ or the oxidation of isoprene emitted from trees.^43^ The photochemistry of pyruvic acid was studied in vapor phase^44^, ^45^ where it was shown that CO_2_ and CH_3_CHO are the major products after irradiation around 340 nm. Studies in aqueous environment^46^ found acetoin, lactic acid and acetic acid being the dominant products after two hours of irradiation.

## Experimental Section

The experimental setup consists of a Bruker APEX Qe Fourier transform ion cyclotron resonance (FT‐ICR) mass spectrometer, equipped with a 9.4 Tesla superconducting magnet, an ESI/MALDI Dual Source II and a Nanobay Console as described previously.^47^ Sodium iodide clusters doped with hydrocarbons are produced by electrospray ionization, transferred through the ion optics and stored in the ICR cell. The ion of interest is coarsely mass selected with a quadrupole mass filter. On‐resonance excitation of all unwanted ions in the ICR cell completes mass selection. The ions are irradiated directly in the ICR cell with light from different tunable laser systems (see Figure S1 for laser power curves). For the UV spectra, an optical parametric oscillator/amplifier EKSPLA NT342B (20 Hz pulse repetition rate), tunable at 225–2600 nm, with typical pulse energies of 3–5 mJ in the UV region is used in the present study. For IR studies in the wavenumber range 2234–3846 cm^−1^, the EKSPLA NT277 laser system (1000 Hz pulse repetition rate) is used with a power range of 25–190 mW. EKSPLA NT273 (1000 Hz pulse repetition rate) enables the measurement of IR spectra in the range of 833–2234 cm^−1^, and emits power of 5–35 mW. Light is admitted to the ICR cell via an electromagnetic shutter, which is, in the case of the 20 Hz system, programmed to open for a defined number of laser pulses. Photofragmentation products are quantified by mass spectrometry. Since the intensity profile of the laser beam in the ICR cell is very homogeneous, absolute absorption cross sections can be derived with the intensity of the fragmentation products. The photodissociation cross section of the precursor ion is calculated as follows [Eq. (1)]:

(1) $$\sigma =\frac{\mathrm{hcA}}{\lambda pE}\left(ln\frac{\sum_{i=0}^{n}I_{i}}{I_{0}}-k_{0}t_{irr}\right)$$

Here, *I*_0_ represents the intensity of the precursor ion after laser irradiation, *I_i_* the intensity of fragment *i*, *h* Planck constant, *c* the speed of light, *A* the area illuminated by the laser beam, *λ* the wavelength, *p* the number of laser pulses, and *E* the energy of a laser pulse. In the case of the measurement with camphor, also fragmentation due to BIRD^48^ (black body infrared radiative dissociation) occurs, *k*_0_ then represents the rate constant for that process and *t*_irr_ the irradiation time with the laser. Since the BIRD process follows an exponential law, the rate can easily be measured.

Absolute IR absorption cross sections are derived from the photofragment intensities by assuming sequential photon absorption following first‐order kinetics. The assumption that the cross section *σ* does not change between the individual absorption events is justified if the clusters do not change structure during heating and if the excited mode lies sufficiently high so that it is not significantly populated after intramolecular vibrational redistribution (IVR). We also assume that radiative cooling is negligible. The ion population is divided into fractions *I_j_* that have absorbed *j* photons. If dissociation occurs upon absorption of the *k*th photon, a set of differential equations describes the evolution of the ion population in time [Eq. (2)]:

(2) $$dI{}_{0}=-I{}_{0}\sigma \Phi dtdI{}_{j}=I{}_{j-1}\sigma \Phi \mathrm{dt}-I{}_{j}\sigma \Phi dt\;\;\;\;\mathrm{for}\;0<j<kdI{}_{k}=I{}_{k-1}\sigma \Phi dt$$

Essentially, this is a series of first‐order reactions with rate coefficient *σΦ*. For comparison with experiment, *I_k_* is the sum of all fragment intensities, and *Φ* is the quasi‐continuous wave photon flux. To obtain *σ_k_* from experiment, we generate a lookup table with intensities *I_k_* as a function of *σΦt*. Together with the experimental parameters *Φ* and *t* this directly yields *σ_k_*. The number of photons *k* required for dissociation was estimated from the theoretically calculated dissociation energy, accounting for the already available thermal energy of the clusters. This estimate may deviate by ±1 from the actual value. In addition, some clusters may need an extra photon because of radiative cooling while others contain thermal energy significantly above average and dissociate one photon earlier. Despite all shortcomings, we believe that this analysis is justified and worthwhile since it provides access to the experimental absorption cross section, with a conservative uncertainty of ±50 %. It also illustrates how the noise level is affected by IRMPD.

In the UV range, the main source of error are the pulse energy fluctuations of the laser system. When these energies were measured repeatedly at 225 nm, a standard deviation of about 0.15 mJ was obtained, at an average pulse energy of 1.77 mJ. Pulse energies in the UV fluctuate significantly since UV photons are generated in four stages of nonlinear optics in the EKSPLA NT342B system, starting from the Nd:YAG pump laser fundamental at 1064 nm. Together with uncertainties in the alignment of the laser beam and beam profile in the ICR cell, at a distance of 3 m from the laser system, the absolute cross sections reported here are estimated to be accurate within 30 %.

All chemicals were purchased from SigmaAldrich with a purity of at least 98 %. In all measurements, a 1:1 mixture of CH_3_OH/H_2_O was used as solvent. The concentration of NaI was held constant at 10 mmol L^−1^. Camphor was added at a concentration of 20 mmol L^−1^, formic and pyruvic acid at 1 mmol L^−1^.

Structures of investigated clusters were optimized at the B3LYP/def2TZVP level of theory. Due to the possible importance of the dispersion forces, we also repeated our calculations including the D2 dispersion correction as introduced by Grimme.^49^ The B3LYP reaction energies for decomposition of NaI clusters were found to be in better agreement with MP2 and CCSD(T)//MP2 results than B3LYP+D2 (see Table S1). Therefore, we used the B3LYP approach for optimizations. Initial structures of Na_*n*_I_*n*−1_^+^ clusters were taken from a previous study of Aguado et al.,^29^ clusters with adsorbed molecules were created by adding the respective species to an optimized cluster. Note that due to the size of the clusters, the found structures do not necessarily represent global minima at the given level of theory. At the B3LYP/def2TZVP level, Na_2_I^+^ is predicted to have a bent structure, in contrast to MP2/def2TZVP or CCSD/def2TZVP calculations that predict a linear one; however, the energy difference between the bent local minimum and the stationary point with imposed linear structure is negligible (0.16 kJ mol^−1^). For calculation of final IR spectra, the most stable clusters found at the B3LYP/def2TZVP level were recalculated at the MP2 level with effective core potentials (ECPs) of ECP10SDF(Na)^50^ and ECP46MDF(I)^51^ (in order to make the calculations more tractable) and def2TZVP basis set for all other atoms, further denoted as “def2TZVP,ECP(Na,I)”. The optimization does not change the structure significantly while the relative energy of isomers might be affected by the relative stability of the NaI cluster structure described with ECPs. Still, the MP2 method is expected to describe the molecule/salt interaction better than DFT, providing more reliable IR spectra. DFT and MP2 IR spectra are in qualitative agreement as shown in the Supporting Information, MP2 spectra reproduce the experimental results more closely. IR spectra were scaled by 0.95 (unless stated otherwise) and artificially broadened by Gaussian functions with a full width at half maximum (FWHM) of 30 cm^−1^.

Excited state properties were calculated by using time‐dependent density functional theory (TDDFT) with BHandHLYP and CAM‐B3LYP functionals, equation of motion—coupled clusters singles and doubles (EOM‐CCSD), and multireference configuration interaction (MRCI). For complexes of NaI salts with molecules, only excited states located predominantly on organic molecules are considered. Benchmark of excited state calculations with respect to various methods and basis sets is provided in the Supporting Information (Table S2). Based on these calculations, we conclude that BHandHLYP results are closer to the EOM‐CCSD results than the ones of CAM‐B3LYP; however, we include results gained using both functionals for comparison. CAM‐B3LYP excitation energies are generally lower and match better to experimental values, probably due to compensation of zero‐point energy effects. Natural transition orbitals^52^ are used for excited states analysis. For the Na_4_I_3_^+^ cluster, spin–orbit coupling within the MRCI method was calculated using the spin–orbit pseudopotential and the state‐interacting method, along with ECPs for Na and I atoms as introduced above (see Table S3 for benchmark calculations).

Due to the presence of radical species with iodide in the cluster, spin–orbit contributions to reaction energies for radical species, as encountered after photodissociation of pyruvate, cannot be excluded. The highest shift can be expected for I^.^, calculated to be −0.30 eV at the MRCI(5,3) level with the ECP basis set. However, these shifts would not affect the conclusions as they make reaction energies less endothermic. For this reason, we neglect the spin–orbit effects within ground‐state calculations.

All calculations were performed in the Gaussian program^53^ with the exception of the EOM‐CCSD and MRCI calculations that were done in the Molpro program.^54^

## Results and Discussion

### Na_*n*_I_*n*−1_^+^ clusters

**Figure 1:**
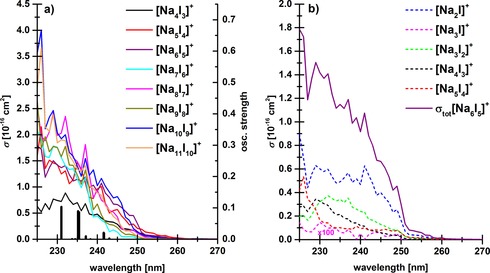
a) Total photodissociation cross section of Na_*n*_I_*n*−1_^+^, *n=*4–11, clusters in the range of 225–260 nm with five laser pulses, and b) fragment resolved photodissociation cross section spectrum of the Na_6_I_5_^+^ cluster. In a), electronic transitions in Na_4_I_3_^+^ calculated at the MRCI(18,12)/ECP10SDF(Na),ECP46MDF(I) level of theory with spin–orbit correlated seven singlet and six triplet states are shown for comparison (black bars).

**Figure 2:**
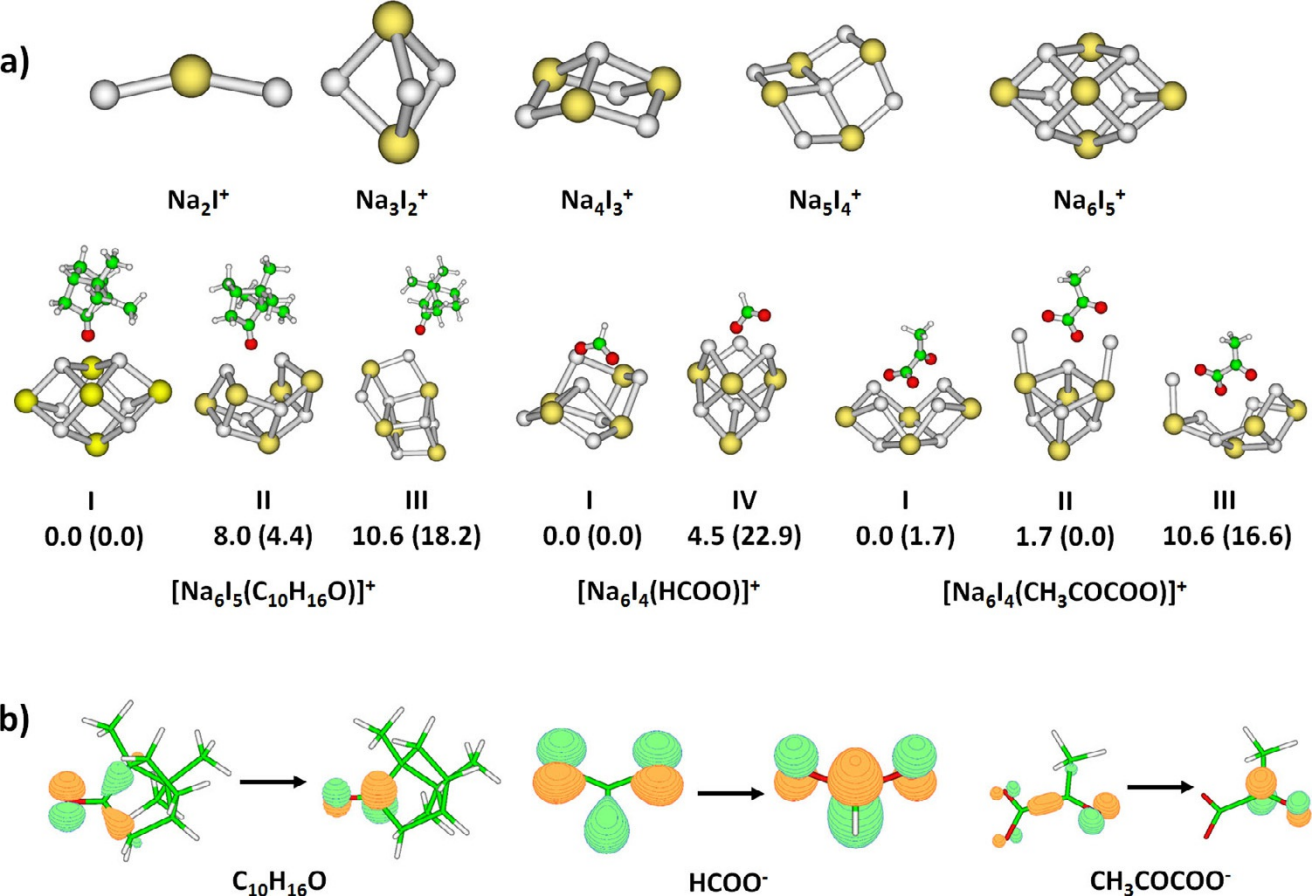
a) Structures of the most stable ions found at the B3LYP/def2TZVP level of theory. For complexes, several isomers with their relative energy [kJ mol^−1^] are shown, with MP2/def2TZVP,ECP(Na,I) energies given in parenthesis. Color code: white: Na, yellow: I, green: C, red: O. b) Character of the first excitation in the respective gas‐phase molecules and ions. Calculated at the TD‐BHandHLYP/def2TZVP level of theory, natural transition orbitals are shown.

Photodissociation spectra of Na_*n*_I_*n*−1_^+^, *n=*4–11, are shown in Figure 1 a, calculated structures of Na_*n*_I_*n*−1_^+^, *n=*2–6, are shown in Figure 2 a. The spectra are very similar, with the absorption starting in all cases around 260 nm towards shorter wavelengths. The absorption maxima are located below 225 nm, outside the wavelength range of the laser. The absorption intensities somewhat increase with cluster size, as expected because the number of chromophores increases. Stoichiometric fragments dominate for all cluster sizes where [NaI]_*x*_ units are evaporated. The non‐stoichiometric Na_3_I^+^ fragment appears for all cluster sizes, most likely formed by dissociation in an excited charge‐transfer state. As the intensity of this fragment is in most cases by two orders of magnitude smaller than the dominant fragments, it is most likely a secondary fragment for the larger clusters (see photodissociation kinetics in Figure S2). After longer irradiation times, significant signal loss occurs, since Na^+^ is formed as secondary fragment from Na_*n*_I_*n*−1_^+^, *n=*2–4, which lies outside the mass range of our FT‐ICR instrument. Therefore, the spectra have been obtained with only five laser pulses, where secondary fragmentation is still negligible. Figure 1 b shows a fragment resolved spectrum for the cluster Na_6_I_5_^+^ with a total cross section around 1.8×10^−16^ cm^−2^ at 225 nm. The absorption of Na_6_I_5_^+^ starts at about 260 nm. For bulk NaI, charge‐transfer transitions are localized in this region.^55^ The spectrum is significantly blue shifted compared to neutral sodium iodide vapor, in which the NaI molecules start absorbing around 400 nm.^56^

For comparison, we also include the calculated photoabsorption spectrum of the Na_4_I_3_^+^ cluster in Figure 1 a. It can be seen that the position of the absorption maximum is well reproduced by our calculations. The particular shape of the spectrum, however, will be subject to considerable thermal broadening. Charge analysis of the respective complete active space self‐consistent field (CASSCF) wave functions using the Mulliken scheme confirms the charge transfer character of the excited states for first six excited states in both singlet and triplet multiplicity, with almost complete electron transfer from I to Na (between 0.92 and 0.99 |*e*| as retrieved by the Mulliken scheme).

### Camphor

**Figure 3:**
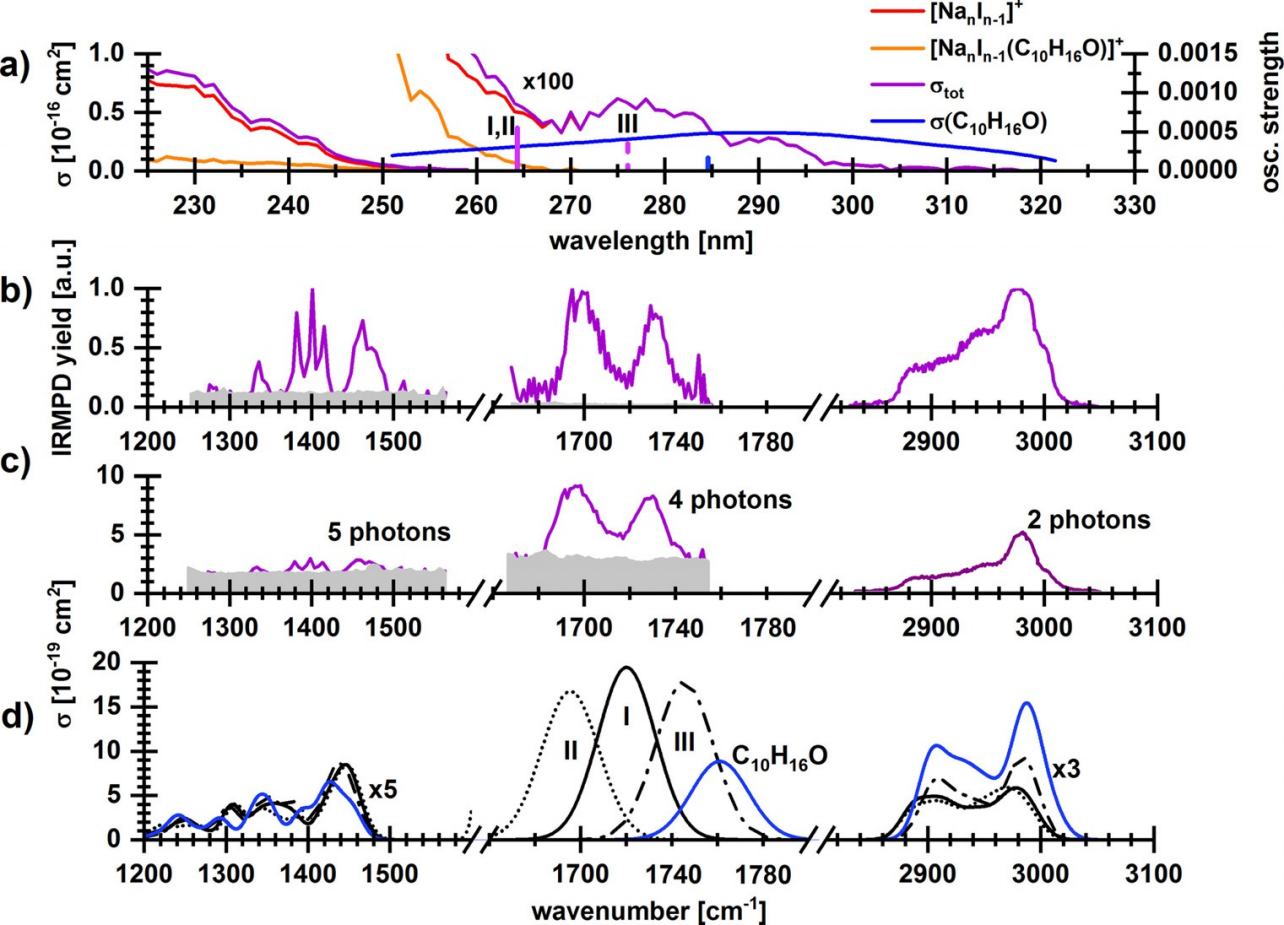
a) Photodissociation spectrum of [Na_6_I_5_(C_10_H_16_O)]^+^ from 225–330 nm applying five laser pulses. The absorption increases with shorter wavelengths and the fragments with the evaporation of [NaI] and [(NaI)_2_(C_10_H_16_O)] are dominant. The absorption cross section of gaseous camphor is also shown.^57^ Transitions calculated at the CAM‐B3LYP/def2TZVP//B3LYP/def2TZVP level of theory for C_10_H_16_O and [Na_6_I_5_(C_10_H_16_O)]^+^ are shown as bars. b) IRMPD spectra recorded at 833–4394 cm^−1^. Here, only the observed absorption bands, 1250–1562, 1666–1754, and 2832–3048 cm^−1^ with an irradiation time of 13, 5, and 3 s, respectively, are shown (noise level in grey). c) IR spectrum retrieved from IRMPD measurement using multiphoton analysis, along with the number of photons used for the analysis. d) Calculated IR spectra at the MP2/def2TZVP,ECP(Na,I) level of theory, wavenumbers were scaled with a factor of 0.95, see Figure S8 for the whole spectrum. For the 1650–1770 cm^−1^ region, the scaling factor of 0.988 was chosen in order to match the experimentally measured C−O frequency of the gas phase camphor at 1762 cm^−1^.^58^ For clarity, the cross section is scaled in the low and high energy part of the spectrum.

The photodissociation spectrum of [Na_6_I_5_(C_10_H_16_O)]^+^ in the range of 225–330 nm is shown in Figure 3 a, Figures S3 and S4 show BIRD and photodissociation kinetics, respectively. Adsorption of camphor on Na_6_I_5_^+^ leads to an extension of the absorption spectrum up to 310 nm, with a local maximum at ≈275 nm. Clearly, camphor acts as the chromophore in this region, albeit two orders of magnitude weaker than the charge transfer excitations of the NaI cluster at shorter wavelengths. Camphor in the gas phase^57^ as well as in aqueous solution (see Figure S5) has an absorption maximum around 290 nm. Adsorption on the cluster leads to blueshift of the maximum by about 15 nm.

To model camphor absorption spectra in clusters, several [Na_6_I_5_(C_10_H_16_O)]^+^ isomers were optimized (Figure S7). As expected, camphor binds to the surface of the salt cluster preferentially through the C−O⋅⋅⋅Na interaction, structures without this pattern are much less stable. In Figure 2 a, we show three selected stable isomers of similar stability, two with C−O interacting with two Na^+^ ions (**I**, **II**) and one in which C−O interacts only with one Na^+^ ion (**III**). In the camphor molecule, the first excitation transition is located on the C−O group (Figure 2 b), we can therefore expect that this interaction affects the excitation energy considerably. In agreement with the experiment, the calculations predict a shift in the position of the first excited state of about +0.2–0.4 eV after camphor adsorption on a salt cluster (Figure 3 a and Table 1). The shift is more pronounced when C−O interacts with two Na^+^ ions. The position of the first excitation in isomer **III** at ≈275 nm suggests that this isomer (or isomers with a similar C−O binding pattern) might be present in the experimental mixture.

**Table 1:** The lowest excitation energies [eV] and oscillator strengths (in parenthesis) of various molecules and salt complexes calculated at the TDDFT level of theory employing the def2TZVP basis set. See the Supporting Information (Table S1) for method benchmark. Structures of ions are included in Figures 2 and S6.

Species	BHandHLYP	CAM‐B3LYP
C_10_H_16_O	4.55 (1.7e‐4)	4.36 (1.7e‐4)
[Na.C_10_H_16_O]^+^	4.79 (4.9e‐4)	4.53 (4.9e‐4)
[Na_2_I.C_10_H_16_O]^+^	4.75 (4.0e‐4)^[a]^	4.51 (4.1e‐4)^[a]^
[Na_3_I_2_.C_10_H_16_O]^+^	4.75 (3.7e‐4)	4.51 (3.7e‐4)
[Na_4_I_3_.C_10_H_16_O]^+^	4.72 (3.8e‐4)	4.48 (3.8e‐4)
[Na_5_I_4_.C_10_H_16_O]^+^	4.74 (3.6e‐4)	4.51 (3.5e‐4)
[Na_6_I_5_.C_10_H_16_O]^+^, **I**	4.94 (5.7e‐4)	4.71 (5.8e‐4)
[Na_6_I_5_.C_10_H_16_O]^+^, **II**	4.94 (5.6e‐4)	4.70 (5.5e‐4)
[Na_6_I_5_.C_10_H_16_O]^+^, **III**	4.74 (3.6e‐4)	4.50 (3.5e‐4)
HCOOH	6.08 (1.3e‐3)	5.89 (1.2e‐3)
HCOO^‐^	6.38 (3.4e‐3); 6.52 (0.0)	6.11 (3.2e‐3); 6.21 (0.0)
CH_3_COCOOH	3.79 (1.5e‐5)	3.58 (1.1e‐5)
CH_3_COCOO^‐^	4.24 (2.4e‐4)	3.97 (5.0e‐4)
[Na_2_.CH_3_COCOO]^+^	4.01 (1.2e‐4)	3.78 (1.3e‐4)
[Na_3_I.CH_3_COCOO]^+^	4.41 (4.5e‐5)	4.18 (4.8e‐5)
[Na_4_I_2_.CH_3_COCOO]^+^	4.08 (1.1e‐4)	3.85 (1.3e‐4)
[Na_5_I_3_.CH_3_COCOO]^+^	3.99 (5.3e‐5)	3.77 (5.4e‐5)
[Na_6_I_4_.CH_3_COCOO]^+^, **I**	4.62 (1.1e‐4)	4.37 (1.9e‐4)
[Na_6_I_4_.CH_3_COCOO]^+^, **II**	4.06 (9.4e‐5)	3.84 (1.0e‐4)

[a] The state is not the lowest excited state in the given geometry, with two electronic transitions among the Na_2_I unit lying below.

Two types of fragmentation are observed in Na_6_I_5_(C_10_H_16_O)^+^: the loss of neutral [NaI]_*x*_ units with camphor remaining on the charged fragment [reaction (1)], and the loss of neutral [NaI]_*x*_ units together with camphor [reaction (2)]. The respective reaction energies are included in Table 2; all of them lie well below the excitation energy.

(1) $$\left[\mathrm{Na}{}_{6}I{}_{5}\left(C{}_{10}H{}_{16}O\right)\right]{}^{+}\to \left[\mathrm{Na}{}_{x}I{}_{x-1}\left(C{}_{10}H{}_{16}O\right)\right]{}^{+}+\left[\mathrm{NaI}\right]{}_{6-x}x=1-5$$

(2) $$\left[\mathrm{Na}{}_{6}I{}_{5}\left(C{}_{10}H{}_{16}O\right)\right]{}^{+}\to \left[\mathrm{Na}{}_{x}I{}_{x\text{-}1}\right]{}^{+}+\left[\mathrm{Na}{}_{6-x}I{}_{6-x}\;\left(C{}_{10}H{}_{16}O\right)\right]x=1-5$$

**Table 2:** Reaction energies [eV] of dissociation channels of various clusters as observed in the experiment (with the exception of the [Na_6_I_5_]^+^ cluster). Calculated at the B3LYP/def2TZVP level.

		*x=*					
Reactants	Products	0	1	2	3	4	5
[Na_6_I_5_]^+^	[Na_6−*x*_I_5−*x*_]^+^ + [NaI]_*x*_	–	1.60	1.31	1.69	1.37	1.96
[Na_6_I_5_(C_10_H_16_O)]^+^	[Na_6−*x*_I_5−*x*_]^+^ + [Na_*x*_I_*x*_(C_10_H_16_O)]_*x*_	1.07	1.91	1.73	2.18	1.87	–
	[Na_6−*x*_I_5−*x*_(C_10_H_16_O)]^+^ + [NaI]_*x*_	–	1.63	1.38	1.66	1.23	1.45
[Na_6_I_4_(HCOO)]^+^	[Na_6−*x*_I_5−*x*_]^+^ + [Na_*x*_I_*x*−1_(HCOO)]_*x*_	–	–	1.40	1.78	1.50	–
	[Na_6−*x*_I_4−*x*_(HCOO)]^+^ + [NaI]_*x*_	–	1.49	1.13	1.55	1.45	–
[Na_6_I_4_(CH_3_COCOO)]^+^	[Na_6−*x*_I_4−*x*_(CH_3_COCOO)]^+^ + [NaI]_*x*_	–	1.56	1.29	1.74	1.27	–
	[Na_6−*x*_I_5−*x*_]^+^ + [Na_*x*_I_*x*−1_(CH_3_COCOO)]	–	2.24	1.70	1.87	1.71	–
	[Na_6−*x*_I_3−*x*_(CH_3_COCOO)]^.+^ + [Na_*x*_I_*x+*1_]^.^	4.40	3.50	4.92	4.25	–	–
	[Na_6−*x*_I_4−*x*_(COO)]^.+^ + Na_x_I_x_CH_3_CO^.^	3.23	4.40	4.23	4.68	–	–
	[Na_6−*x*_I_4−*x*_O]^.+^ + [NaI]_*x*_.CH_3_COCO^.^	–	–	5.95	6.46	–	–
	[Na_6−*x*_I_4−*x*_]^.+^ + [NaI]_*x*_⋅CH_3_COCO_2_^.^	6.05	5.76	5.09	5.03	–	–

It is immediately evident from Figure 3 a that when camphor acts as a chromophore, reaction (2) dominates by far, the ionic products consist mostly of Na_4_I_3_^+^. Thus, we can expect that, after excitation into the first excited state, internal conversion to the ground state takes place. The photon energy (about 4.3 eV) is then converted into vibrational degrees of freedom of [Na_6_I_5_(C_10_H_16_O)]^+^, followed by statistical decomposition. Calculations show that the statistically most probable dissociation channel in the ground state is loss of the camphor unit, with an energy of 1.07 eV (Table 2). The energy remaining in the cluster is then sufficient to cause further evaporation of the Na_6_I_5_^+^ cluster, with reaction energies of all primary decomposition reactions producing Na_*n*_I_*n*−1_^+^ and a neutral Na_*x*_I_*x*_ unit lying below 2 eV (Table 2).

Below 280 nm, where Na_6_I_5_^+^ absorption sets in, the dominant fragment is still Na_4_I_3_^+^, followed closely by [Na_5_I_4_(C_10_H_16_O)]^+^, which is formed by loss of a single NaI molecule, with a calculated reaction energy of 1.63 eV. Below 260 nm, more than 3 eV extra energy is available to cause further dissociation, but camphor may stay on the cluster. In this region, the fragmentation behavior is similar to [Na_*n*_I_*n*−1_(CH_3_OH)]^+^ studied previously by Misaizu et al.^31^ Together with the fact that camphor is preferentially evaporated at longer wavelengths, this clearly points to non‐statistical dissociation of [Na_6_I_5_(C_10_H_16_O)]^+^ after an excitation within the NaI cluster, most likely taking place in an excited state. Photodissociation of camphor itself, that is bond cleavage within the camphor moiety, occurred neither after excitation of the molecule nor of the salt cluster.

Figure 3 b shows the measured IRMPD spectrum and Figure 3 c the reconstructed one‐photon spectrum in absolute units. The loss of camphor leads to Na_6_I_5_^+^, which is the only fragment observed at 1250–1562 cm^−1^. The estimated number of photons needed for dissociation (five) explains the relative high noise level in the reconstructed spectrum. In the 1666–1754 cm^−1^ region, the intense C−O vibration leads to appreciable fragment signal. Here, also other pure fragments Na_*n*_I_*n*−1_^+^, *n=*2, 4, are observed with small intensities. The shift of ≈30–60 cm^−1^ with respect to the camphor C−O vibration position in the gas phase (1762 cm^−1^)^58^ suggests a strong interaction with the salt. About two photons are required for the evaporation of camphor in the 2832–3048 cm^−1^ region of C−H vibrations, which together with the higher laser power provides a high signal‐to‐noise ratio. All spectral features have similar dissociation cross section of the order of 10^−19^ cm^2^.

Figure 3 d and Figure S8 show calculated IR spectra of various [Na_6_I_5_(C_10_H_16_O)]^+^ isomers as well as of the C_10_H_16_O molecule. When camphor is adsorbed on the Na_6_I_5_^+^ cluster through the C−O bond, only minor changes are observed in 1200–1600 cm^−1^ and 2800–3100 cm^−1^ regions where various C−C and C−H vibrations are IR‐active. When the C−O⋅⋅⋅Na interaction is missing, as in the high‐energy isomer **VIII** (see Figure S8), the spectrum in the 2800–3100 cm^−1^ region shifts to lower wavenumbers due to C−H bond interaction with the cluster, which is not observed in the experiment. Our calculations also show that the shape of the band at 3000 cm^−1^ results from the convolution of all C−H stretching modes, with no prominent absorptions of high intensity.

In the 1700–1750 cm^−1^ region, two IR peaks separated by about 30 cm^−1^ were found in the experiment. In calculations, a specific multiplication factor had to be included in order to reproduce the experimental position of the C−O vibration in the gas phase camphor molecule,^58^ indicating that we might reach the limits of our computational approach here. IR peaks of isomers **I**–**III** are separated by about 25 cm^−1^, reproducing the experimentally recorded shift, their position makes it however difficult to decide which isomers are present in the experimental mixture. As expected, the interaction of the carbonyl oxygen with two Na^+^ ions in more stable isomers **I**, **II** leads to a stronger redshift with respect to isomer **III** with weaker interaction.

Our analysis of UV and IR spectra has shown that isomers with camphor located on the surface of the NaI salt cluster, in which the carbonyl oxygen interacts with one or two Na^+^ ions, are the dominant structural motif, with at least two isomers coexisting in the experimental mixture. Calculations could also reproduce the absolute intensity of reconstructed one‐photon IR spectra (within the factor of ≈3).

### Formic acid

**Figure 4:**
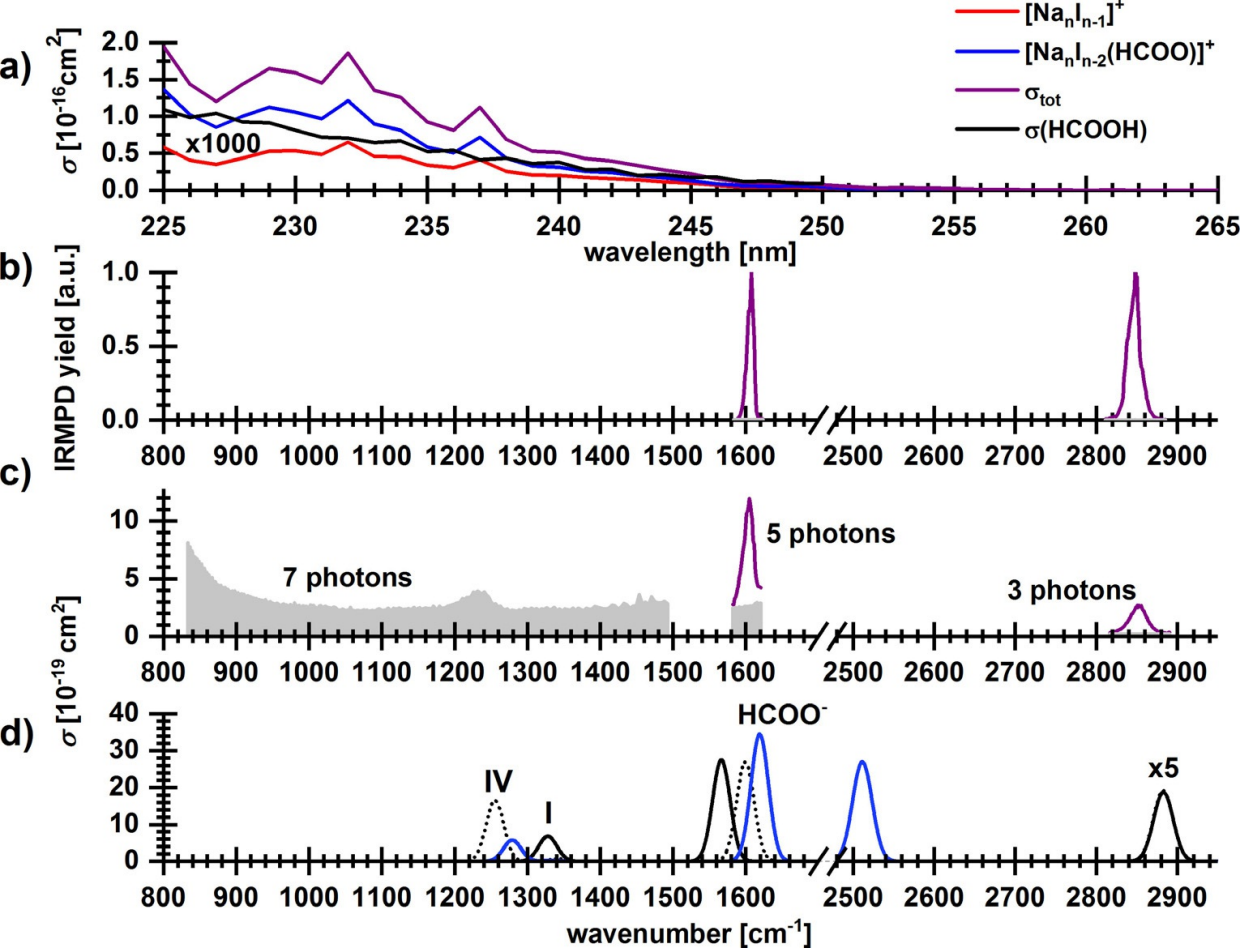
a) Photodissociation spectrum of [Na_6_I_4_(HCOO)]^+^ from 225–260 nm and five laser pulses. Gas phase absorption cross section *σ* of neutral formic acid (HCOOH) is also shown.^59^ Note that theoretically calculated transitions lie outside the depicted region (Table 1). b) IRMPD spectral bands observed at 952–1492, 1582–1620 and 2816–2890 cm^−1^ with 10, 5 and 3 s irradiation time, respectively (noise level in grey). c) IR spectrum retrieved from IRMPD measurement using multiphoton analysis, along with the number of photons used for the analysis. d) Calculated IR spectra at the MP2/def2TZVP,ECP(Na,I) level of theory, wavenumbers were scaled with a factor of 0.95, see Figure S10 for the whole spectrum. For clarity, the cross section of **I** and **IV** is scaled in the high‐energy part of the spectrum.

Figure 4 a shows the total and fragment resolved photodissociation spectrum of [Na_6_I_4_(HCOO)]^+^ in the range of 225–260 nm and the absorption cross section of neutral formic acid in the gas phase.^59^ Formic acid and formate are calculated to absorb at 6.0–6.4 eV (Table 2). The calculated excitation energy of HCOOH (5.9–6.1 eV, depending on the method) lies close to the one measured by Singleton et al., ≈5.8 eV.^59^ Deprotonation is calculated to induce a shift of the first excitation energy of about +0.3 eV. This excitation is delocalized over the whole HCOO^−^ ion (Figure 2 b) and can be expected to shift considerably with changes in the environment.

The absorption cross section of formic acid lies in the same energy range as absorption of Na_*n*_I_*n*−1_^+^ clusters and is two orders of magnitude less intense the one for Na_*n*_I_*n*−1_^+^. Therefore, electronic excitations of the formate anion are not expected to contribute much to the total cross section of the investigated [Na_6_I_4_(HCOO)]^+^ cluster, the spectrum being dominated by charge‐transfer transitions within the salt cluster. From the theoretical perspective, transitions taking place on HCOO^−^ within the [Na_*x*_I_*x*−2_(HCOO)]^+^ clusters cannot be easily distinguished and are not included in Table 1.

The two main fragmentation channels are loss of [NaI]_*x*_ and Na_*x*_I_*x*−1_HCOO:

(3) $$\left[\mathrm{Na}{}_{6}I{}_{4}\left(\mathrm{HCOO}\right)\right]{}^{+}\to \left[\mathrm{Na}{}_{6-x}I{}_{4-x}\left(\mathrm{HCOO}\right)\right]{}^{+}+\left[\mathrm{NaI}\right]{}_{x}x=1-4$$

(4) $$\left[\mathrm{Na}{}_{6}I{}_{4}\left(\mathrm{HCOO}\right)\right]{}^{+}\to \left[\mathrm{Na}{}_{6-x}I{}_{5-x}\right]{}^{+}+\left[\mathrm{Na}{}_{x}I{}_{x-1}\left(\mathrm{HCOO}\right)\right]x=2-4$$

All reactions require less than 2 eV of energy (Table 2). The kinetics (see Figure S9) clearly show that the radical products [NaI]^.+^, [Na_3_I]^.+^ and [Na_3_I(COO)]^.+^ are secondary products. Due to their overall low intensities, their precursors cannot be identified.

Figure 4 b, c shows the observed IRMPD bands of [Na_6_I_4_(HCOO)]^+^ and the respective reconstructed spectrum for one photon absorption, respectively. One‐photon spectra predict less sharp peaks compared to the measured IRMPD data. At 1582–1620 cm^−1^, the COO group absorbs, with a relatively narrow peak at ≈1600 cm^−1^. The most intense and most favorable fragmentation channel (see Table 2), is the evaporation of Na_2_I_2_ with 1.13 eV, which requires nominally 5.7 photons. Another fragmentation channel is observed to be the evaporation of a single NaI unit, which requires 1.49 eV or nominally 7.5 photons at 1600 cm^−1^. At 2820–2880 cm^−1^, the C−H bond absorbs, with a peak width of 15 cm^−1^. The dominant neutral fragment here is Na_2_I_2_, which is energetically accessible with absorption of 3.2 photons at 2850 cm^−1^.

Calculated IR spectra of [Na_6_I_4_(HCOO)]^+^ are shown in Figure 4 d. We localized six isomers with different bonding patterns (Figure S7) and selected two isomers that have a different manner of HCOO^−^ binding to the cluster, either with both C−O bonds (**I**) or predominantly with one of them (**IV**). When HCOO^−^ is embedded in a salt cluster, the position of the C−H vibration shifts considerably to higher wavenumbers, resembling more closely the situation in the neutral formic acid. Almost all investigated isomers have virtually the same IR spectrum in the ≈2850 cm^−1^ region as the C−H bond is nearly unaffected by the interaction with the salt (see Figure S10). In the low‐energy region, on the other hand, the IR spectrum is considerably affected by interaction of C−O bonds with the salt cluster. Here, the spectrum of isomer **IV** interacting with the salt cluster only through one C−O bond lies closest to the experimentally measured peak at ≈1600 cm^−1^ (see Figures 4 d and S10). However, position of the C−O band in isomer **I** is still relatively close (≈1570 cm^−1^) and, based on analogy with camphor, it cannot be excluded that this isomer is the dominant one. For both experimentally observed peaks, absolute cross section of the reconstructed IR spectrum in Figure 4 c is well reproduced by theoretical calculations.

There is one more important aspect of the IRMPD spectrum. Calculations in Figure 4 d predict that there are two peaks arising from the COO stretching vibrations, a more intense peak at ≈1600 cm^−1^ due to the antisymmetric and a less intense one at ≈1250 cm^−1^ corresponding to the symmetric stretching mode. However, only the peak at 1600 cm^−1^ is experimentally observed. In Figure 4 c, we account for multiphoton processes under the assumption that seven photons are needed in the 800–1500 cm^−1^ region (the lowest lying dissociation channel leading to [Na_4_I_2_(HCOO)]^+^ and Na_2_I_2_ requires about 1.1 eV, see Table 2). Then, noise level of ≈3×10^−19^ cm^2^ can be expected. The peak at ≈1250 cm^−1^ might therefore disappear in noise, depending on its experimental width and details of the IRMPD process.

### Pyruvic acid

**Figure 5:**
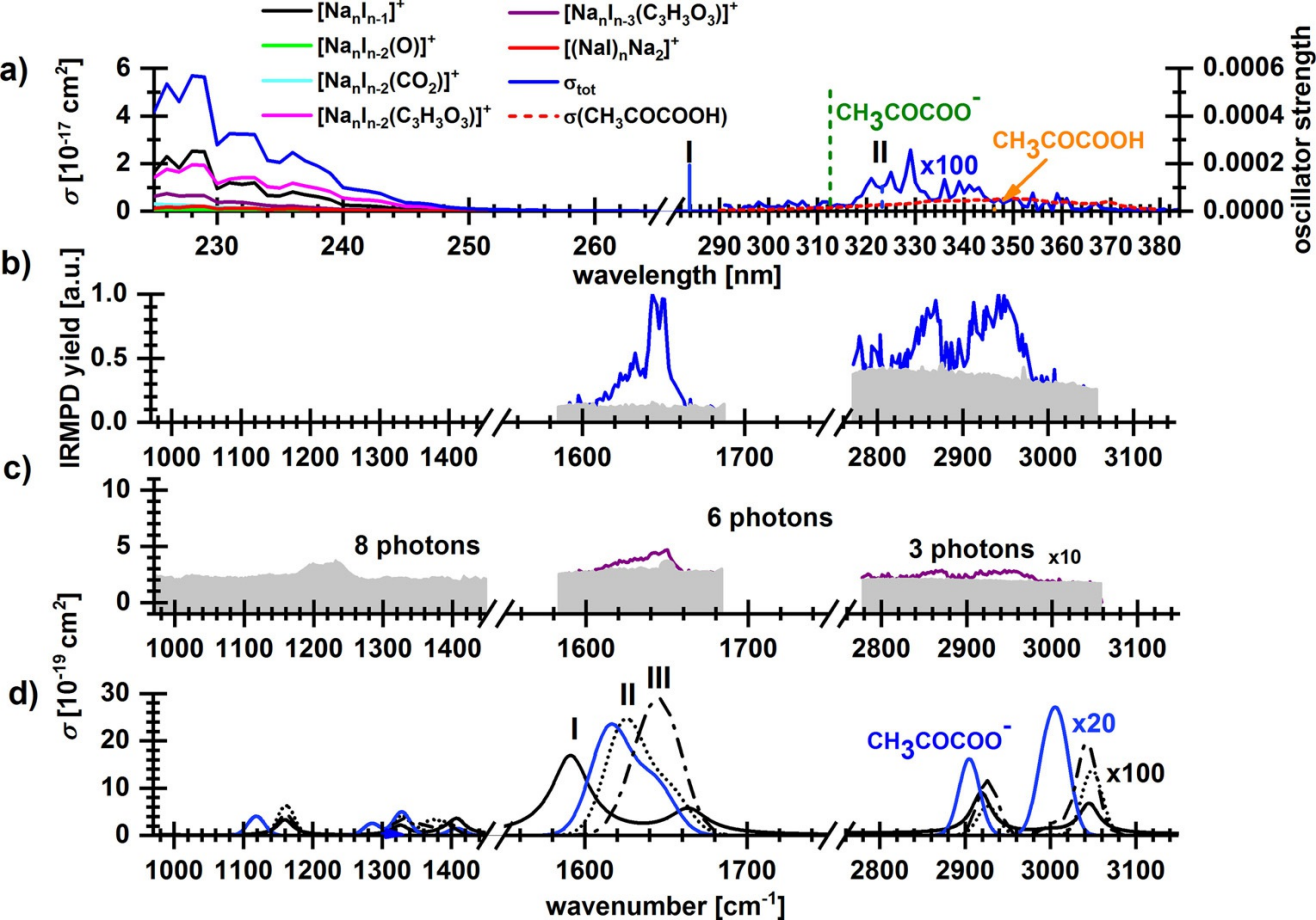
a) Total photodissociation cross section of [Na_6_I_4_(CH_3_COCOO)]^+^ and the contribution of the fragments at 225–389 nm and 20 pulses. Absorption cross sections of neutral gaseous pyruvic acid are also shown.^60^ Transitions calculated at the CAM‐B3LYP/def2TZVP//B3LYP/def2TZVP level of theory for CH_3_COCOOH, CH_3_COCOO^−^ and [Na_6_I_4_(CH_3_COCOO)]^+^ are shown as bars. b) IRMPD spectrum of the same precursor ion at 970–1470, 1583–1683 and 2777–3058 cm^−1^ with irradiation time of 17, 10 and 4 s, respectively (noise level in grey). c) IR spectrum retrieved from IRMPD measurement using multiphoton analysis, along with the number of photons used for the analysis. d) Calculated IR spectra at the MP2/def2TZVP,ECP(Na,I) level of theory, wavenumbers were scaled with a factor of 0.95, see Figure S11 for the whole spectrum. For clarity, the cross section is scaled in the high‐energy part of the spectrum.

Figure 5 a shows the total photodissociation cross section measured for [Na_6_I_4_(CH_3_COCOO)]^+^ at 225–389 nm, together with literature data^43^, ^60^ for neutral pyruvic acid in the gas phase. The absorption for wavelengths >260 nm can be clearly attributed to pyruvate, with a discrete structure. The total measured cross section is similar to the gas‐phase spectra of CH_3_COCOOH from literature measured at 0.9 mbar^43^ and 0.27–1.3 mbar,^60^ respectively, with a blue‐shift of 20–25 nm.

Excited state calculations (Table 1) can reproduce the experimental observation. Pyruvic acid absorbs at about 3.7 eV, deprotonation shifts the first excitation energy by about +0.4 eV. Complexation with the cluster induces a shift backwards by −0.2 eV, which can be rationalized by the fact that the first electronic transition in CH_3_COCOO^−^ is located on the central C−O group (Figure 2 b) that takes part in complexation with the salt. Note however that the excitation energy depends also sensitively on the cluster structure: for [Na_6_I_4_(CH_3_COCOO)]^+^, the excitation energy of the almost isoenergetic isomers **I** and **II** (Figure 2) differs by 0.6 eV. The absorption of isomer **I** might therefore be hidden within the onset of the NaI absorption.

The energetics of various dissociation channels is included in Table 2. The dominant fragmentation pathway in the 291–389 nm region is loss of neutral [NaI]_*x*_:

(5) $$\mathrm{Na}{}_{6}I{}_{4}\left(\mathrm{CH}{}_{3}\mathrm{COCOO}\right){}^{+}\to \mathrm{Na}{}_{6-x}I{}_{4-x}\left(\mathrm{CH}{}_{3}\mathrm{COCOO}\right){}^{+}+\mathrm{Na}{}_{x}I{}_{x}x=2-4$$

The first absorption feature appears slightly below 370 nm, which corresponds to 3.4 eV. Also, pure sodium iodide clusters Na_*x*_I_*x*−1_^+^ are observed as minor products. The dominance of Na_6−*x*_I_4−*x*_(CH_3_COCOO)^+^ indicates that internal conversion is followed by intramolecular vibrational redistribution, leading to statistical dissociation of the cluster. This is corroborated by the observation that the Na_3_I(CH_3_COCOO)^+^ fragment, which according to the calculations requires about 0.4 eV more energy than the other two cluster sizes, is observed only below 350 nm. No photolysis of pyruvate is observed within the tropospherically relevant wavelength region above 290 nm, although the direct C−C bond photolysis would be energetically accessible below 365 nm. However, the experimental noise level yields an upper limit of 2×10^−20^ cm^2^ for the photolysis cross section of pyruvate embedded in the salt cluster in the actinic region. This means that photolysis products formed with similar cross sections as recently observed for glyoxylate^32^ would be hidden in the noise.

At 225–260 nm, the total cross section increases to about 10^−17^ cm^2^. Here, loss of Na_*x*_I_*x*_ is observed, with *x=*1–3. In addition, pure sodium iodide clusters Na_6−*x*_I_5−*x*_^+^ are observed:

(6) $$\mathrm{Na}{}_{6}I{}_{4}\left(\mathrm{CH}{}_{3}\mathrm{COCOO}\right){}^{+}\to \mathrm{Na}{}_{6-x}I{}_{5-x}{}^{+}+\left[\mathrm{Na}{}_{x}I{}_{x-1}\left(\mathrm{CH}{}_{3}\mathrm{COCOO}\right)\right]{}_{x}x=1-4$$

A pronounced frequency dependence of the two fragment types is observed. Fragmentation following reaction (6) dominates below 230 nm, while reaction (5) dominates above. Both reactions are calculated to have dissociation energies below 2.5 eV (Table 2).

Non‐stoichiometric fragments are also observed, namely loss of a neutral iodine atom, which may be embedded in a neutral sodium iodide cluster, reaction (7), and photolysis of the pyruvate C−C bond, reaction (8). The latter reaction leads to formation of a carbon dioxide radical anion CO_2_^.−^ stabilized by the salt environment, similar to our previous study of glyoxylate photolysis in sodium chloride clusters.^32^ Both reactions require more energy than reactions (5) and (6), but are very well accessible under the given conditions (Table 2).

(7) $$\left[\mathrm{Na}{}_{6}I{}_{4}\left(\mathrm{CH}{}_{3}\mathrm{COCOO}\right)\right]{}^{+}\to \left[\mathrm{Na}{}_{6-x}I{}_{3-x}\left(\mathrm{CH}{}_{3}\mathrm{COCOO}\right)\right]{}^{•}{}^{+}+\left[\mathrm{Na}{}_{x}I{}_{x+1}\right]{}^{•}x=0-3$$

(8) $$\left[\mathrm{Na}{}_{6}I{}_{4}\left(\mathrm{CH}{}_{3}\mathrm{COCOO}\right)\right]{}^{+}\to \left[\mathrm{Na}{}_{6-x}I{}_{4-x}\left(\mathrm{COO}\right)\right]{}^{•}{}^{+}+\mathrm{Na}{}_{x}I{}_{x}\mathrm{CH}{}_{3}\mathrm{CO}{}^{•}x=0-3$$

The carbon dioxide radical as well as (CO_2_)_*n*_^.−^ clusters have been already observed in spectroscopy experiments^32^, ^61^, ^62^, ^63^, ^64^, ^65^, ^66^, ^67^, ^68^, ^69^, ^70^ and can be stored in the ICR cell on the timescale of seconds.

Additional weak fragment ions are found, namely [Na_*x*_I_*x*−2_O]^.+^, *x=*3–4, and Na_*x*_I_*x*−2_^.+^, *x=*3–6. The fragment series [Na_*x*_I_*x*−2_O]^.+^ is not observed at low pulse numbers, which clearly indicates that these are formed in secondary reactions. We expect that these arise as secondary products from the [Na_*x*_I_*x*−2_COO]^.+^ fragments, through elimination of CO as observed in gas‐phase studies of CO_2_^.−[64, 71]^ [reaction (9)] or via a charge transfer transition from CO_2_^.−^ to Na^+^ [reaction (10)]. The photochemistry of pyruvate embedded in the cluster differs from the photolysis of pyruvic acid,^44^, ^45^, ^46^ partly because the acid proton is not available for rearrangements.

(9) $$\left[\mathrm{Na}{}_{x}I{}_{x-2}\mathrm{COO}\right]{}^{+}\to \left[\mathrm{Na}{}_{x-y}I{}_{x-y-2}O\right]{}^{+}+\left[\mathrm{NaI}\right]{}_{y}\cdot \mathrm{CO}x=3-6,\;y=0-3$$

(10) $$\left[\mathrm{Na}{}_{x}I{}_{x-2}\mathrm{COO}\right]{}^{+}\to \left[\mathrm{Na}{}_{x-y}I{}_{x-y-2}\right]{}^{+}+\left[\mathrm{NaI}\right]{}_{y}\cdot \mathrm{CO}{}_{2}x=3-6,\;y=0-3$$

Figure 5 b shows the measured IR bands and Figure 5 c the reconstructed one‐photon spectrum. At 1583–1683 cm^−1^, C−O vibrations originating from the carbonyl as well as carboxyl groups induce the fragmentation mainly into [Na_2_(CH_3_COCOO)]^+^. Calculations confirm that this is the fragmentation pathway with the lowest reaction energy (Table 2). Another observed channel is evaporation of one NaI unit. The strongest feature at 1640 cm^−1^ may result from carbonyl groups that are weakly coupled to the salt cluster, while the antisymmetric stretching mode of the carboxyl group may be responsible for the broader feature on the low‐energy side of the peak. In the region of the C−H vibrations, 2775–3050 cm^−1^, the dominant fragment is the evaporation of Na_2_I_2_. This is almost isoenergetic with the evaporation of Na_4_I_4_, the dissociation channel with the smallest reaction energy. These IR bands have only low intensity and are almost lost in the noise level in the reconstructed one‐photon spectrum.

Figure 5 d shows computed IR spectra of three selected clusters, with different degree of pyruvate‐salt interaction, isomers **I** and **III** being more incorporated into the cluster compared to isomer **II**. In the high‐energy region where C−H bonds absorb, two distinct peaks of very low intensity are observed, in agreement with the experiment. However, we were unable to reproduce their position quantitatively. Position of the measured bands seem to agree well with the one of gas‐phase pyruvate. A shift of +20–50 cm^−1^ is induced upon adsorption on a salt cluster, away from the experimental spectrum. This spectral characteristics is common for all found isomers (Figure S7) at both DFT and MP2 level and the shift is observed already in the [Na_2_(CH_3_COCOO)]^+^ cluster (Figure S12). We therefore conclude that our calculations are systematically shifted in this region. In the low‐energy part, calculations predict intense absorptions in the 1600–1700 cm^−1^ region, in agreement with the experiment, with isomers **II** and **III** well reproducing the measured spectrum. Note that two intense peaks that contribute to this band are well separated at the DFT level but merge for some isomers at the MP2 level. There is also no clear pattern with respect to the spectrum–structure relationship. Finally, we note that the calculated absorption at 1300–1450 cm^−1^ was not observed in the experiment, again because of the IRMPD detection scheme as discussed above for the formate ion, here with the expected noise level of ≈3×10^−19^ cm^2^.

## Conclusions

We investigated photodissociation of three different hydrocarbons embedded in sodium iodide clusters from the UV to the MID‐IR region of the electromagnetic spectrum, 225–12 000 nm. The structural motifs were identified by comparison of UV and IR spectra with quantum chemical calculations. Camphor binds via its carbonyl group, which interacts with one or two sodium ions, while the deprotonated acids are incorporated in the salt structure, replacing iodide. Adsorption on the cluster takes place through photochemically active groups, inducing a shift in absorption spectra with respect to the corresponding gas‐phase molecules. Two common photofragmentation channels were observed. The first one is evaporation of [NaI]_*x*_ units, the second was found to be the evaporation of sodium iodide together with the hydrocarbon. Pyruvate is the only hydrocarbon studied that undergoes photolysis as a primary reaction, with C−C bond cleavage producing a CO_2_^.−^ radical stabilized by the interaction with sodium ions, for wavelengths below 260 nm. This stabilization of CO_2_^.−^ was also observed in our earlier study of glyoxylate embedded in sodium chloride clusters.

We reconstructed one‐photon spectra from the IRMPD measurements and reached semi‐quantitative agreement with calculated absolute cross sections. By analysis of UV and IR spectra, we can conclude that above 260 nm, the organic moiety is the chromophore. After excitation of the hydrocarbon, the energy is redistributed over all degrees of the freedom of the cluster, inducing its decomposition. Our experiment has shown that within the tropospherical absorption region above 290 nm, excitation of the hydrocarbons adsorbed on sodium iodide salts does not lead to photochemical reaction products of the organic moieties in the present study, with the salt cluster environment providing a possibility for efficient disposal of the excess energy. This suggests that the photochemical lifetime of pyruvate may be increased in sea‐salt aerosols, compared to the gas phase. Also, the presence of iodide does not seem to cause any specific photochemistry involving the hydrocarbon moieties.

## Conflict of interest

The authors declare no conflict of interest.

## Biographical Information

*Milan Ončák is a theoretical chemist specialized in computational photochemistry and photodynamics. He received his Ph.D. in 2013 at the University of Chemistry and Technology, Prague, under the supervision of Prof. Petr Slavíček. After spending three years at the Humboldt University of Berlin with Prof. Joachim Sauer as an Alexander von Humboldt Fellow, he moved in 2016 to the group of Prof. Martin K. Beyer at the University of Innsbruck with a Lise Meitner Fellowship funded by the Austrian Research Fund FWF. The cooperation with the experimental hosting group includes, inter alia, topics of aerosol photochemistry, photocorrosion studies on hydrated metal ions and polymer mechanochemistry*.

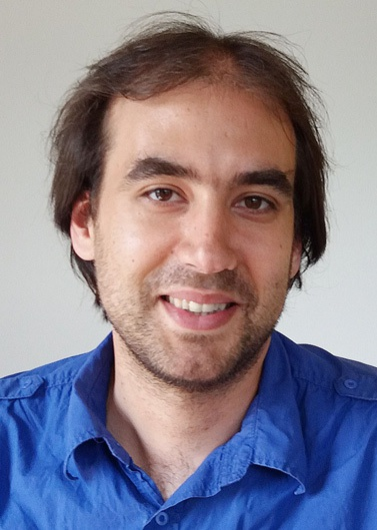

## Supporting information

As a service to our authors and readers, this journal provides supporting information supplied by the authors. Such materials are peer reviewed and may be re‐organized for online delivery, but are not copy‐edited or typeset. Technical support issues arising from supporting information (other than missing files) should be addressed to the authors.

Supplementary
