# Supplementary material for: Photodissociation of Sodium Iodide Clusters Doped with Small Hydrocarbons
Source: Chemistry. 2018 Jul 27;24(47):12433–43. doi: 10.1002/chem.201803017 (PMC6120481; doi:10.1002/chem.201803017)
Supplement: Supplementary file 1 — Supplementary [file CHEM-24-12433-s001.pdf]

CHEMISTRY
A European Journal

Supporting Information

Photodissociation of Sodium Iodide Clusters Doped with Small Hydrocarbons

Nina K. Bersenkowitsch, Milan Ončák,* Jakob Heller, Christian van der Linde, and Martin K. Beyer*[a]

chem_201803017_sm_miscellaneous_information.pdf

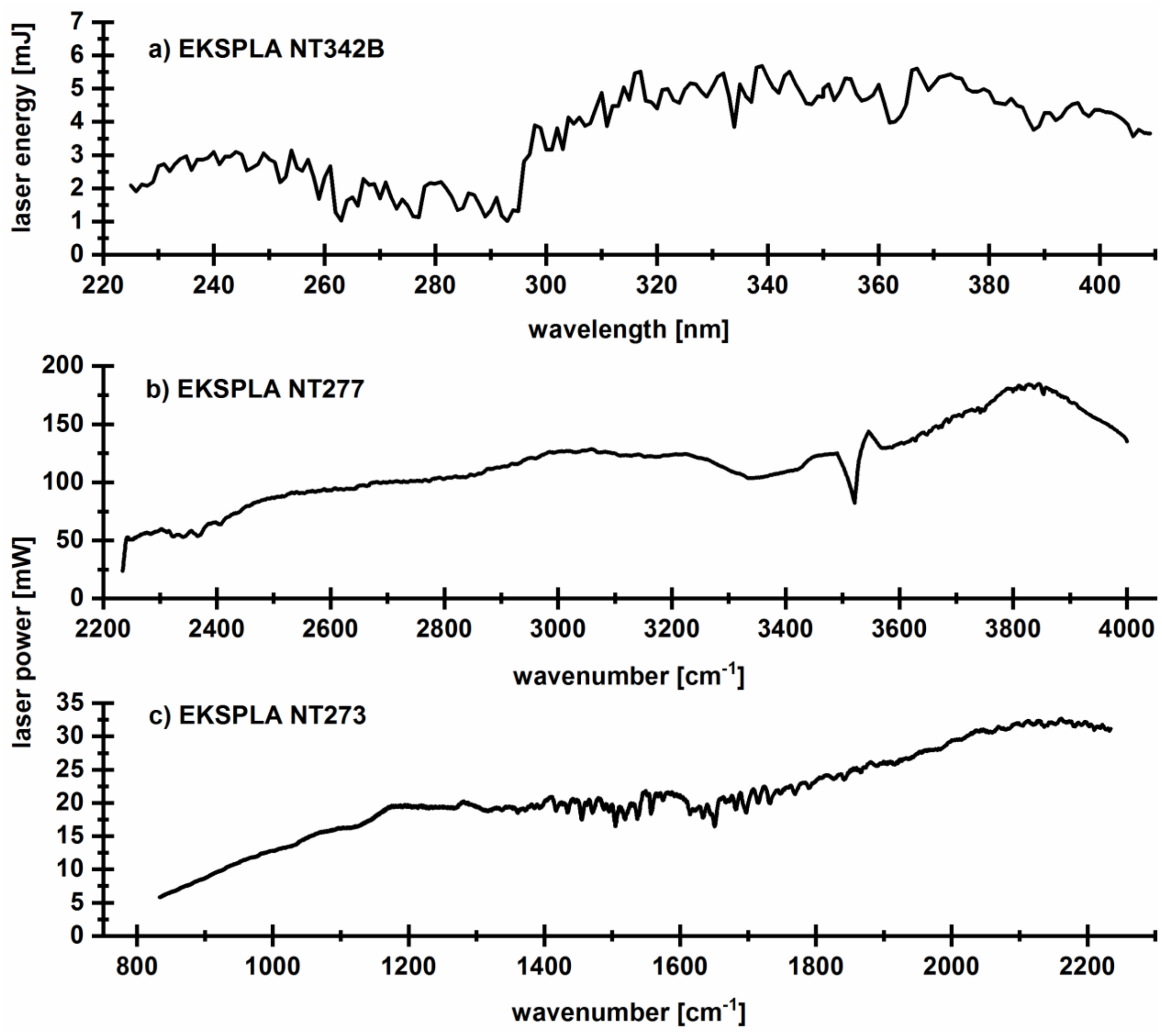

Figure S1: Laser energies for all used tunable laser systems. a) and c) were measured directly in front of the laser, while b) was measured at about 2m distance.

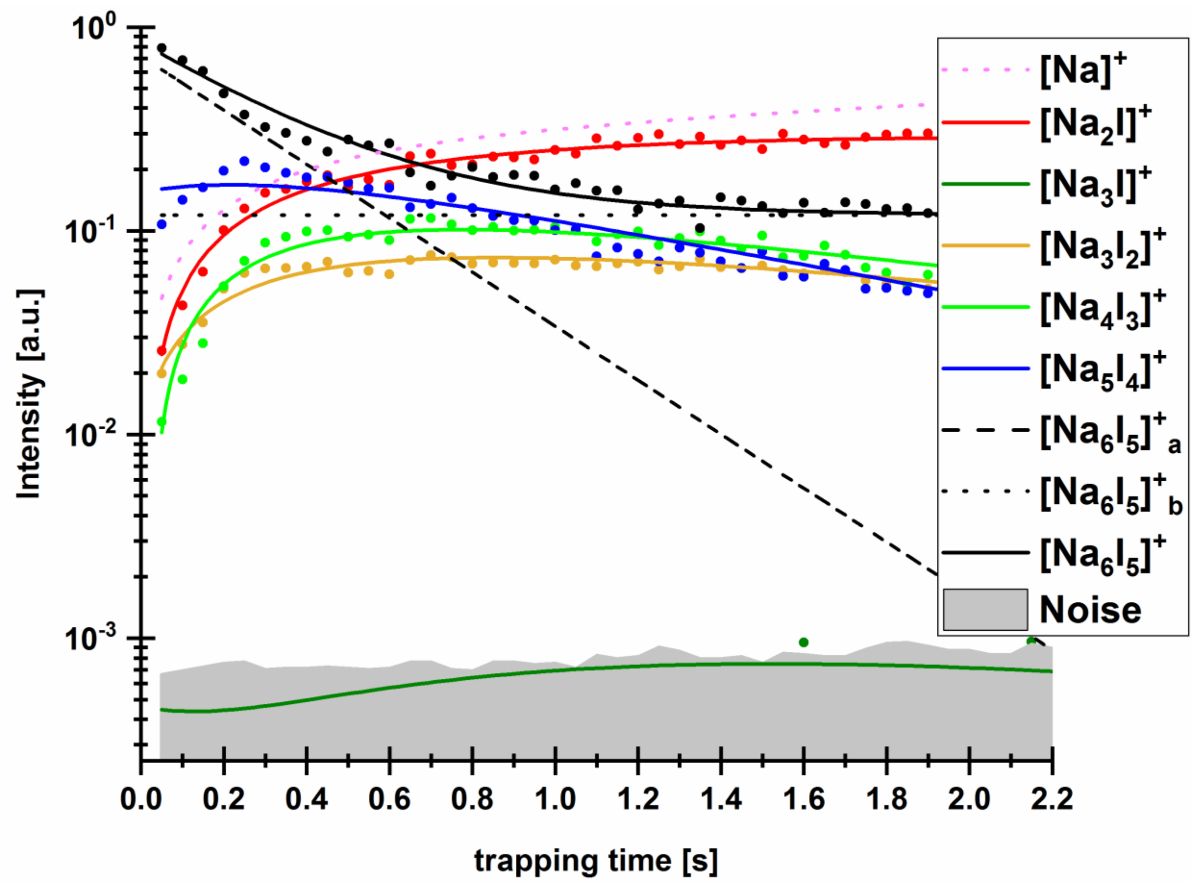

Figure S2: Kinetics of $[Na_6I_5]^+$ at 225 nm with a trapping time of 0-2.2 s. Na^+ lies outside mass window of the instrument and was fitted as a dark channel.

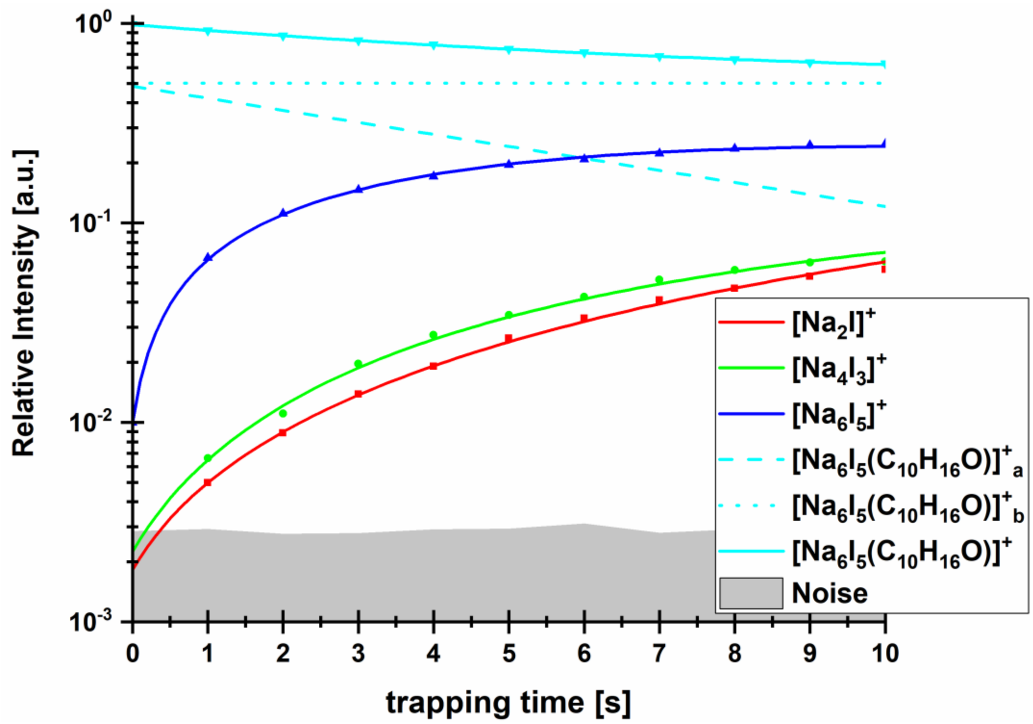

Figure S3: BIRD measurement of the cluster $[Na_6I_5(C_{10}H_{16}O)]^+$. The ions are stored in the cell without laser irradiation and the intensities of the fragments were measured as a function of trapping time. The dashed line represent the fraction of the precursor cluster that fragments due to background radiation, while the dotted line shows the amount of non-fragmenting clusters.

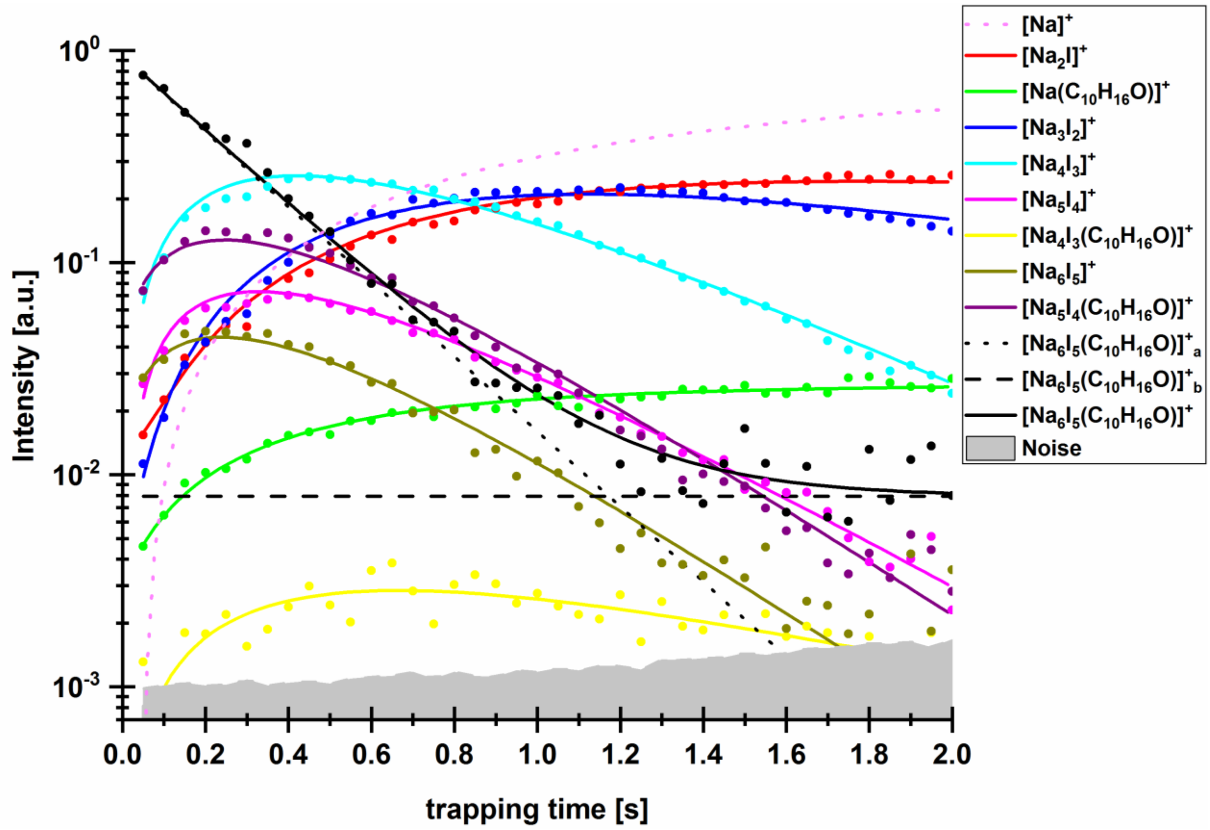

Figure S4: Kinetics of $[Na_6I_5(C_{10}H_{16}O)]^+$ at 225 nm with 0-2 s trapping time. Na^+ lies outside mass window of the instrument and was fitted as a dark channel.

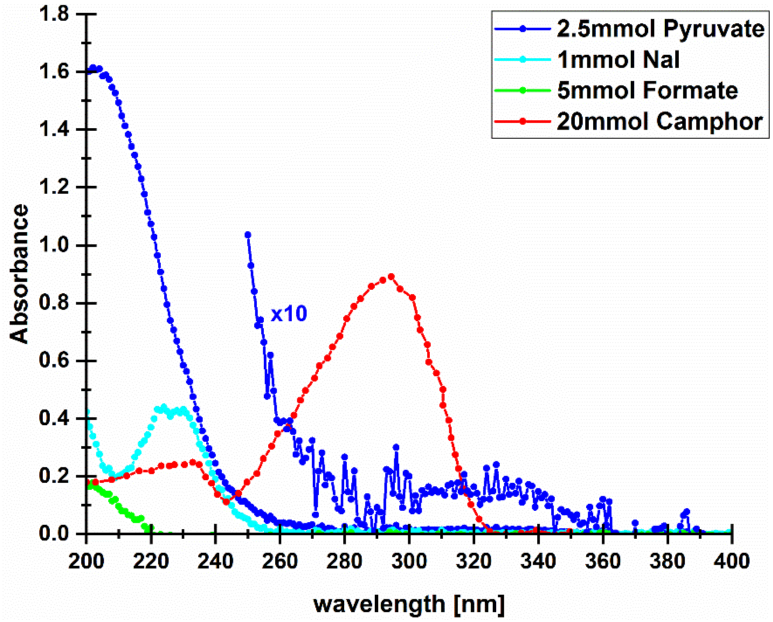

Figure S5: The absorption of 20 mmol camphor, 5 mmol formate, 2.5 mmol pyruvate and 1 mmol NaI were measured in aqueous solution with the Genova Plus UV/VIS spectrophotometer. While the absorption of the formate is completely out of the laser coverage, all other measured substances absorb from 225 nm to longer wavelengths. Camphor shows an absorption peak from 240 – 325 nm.

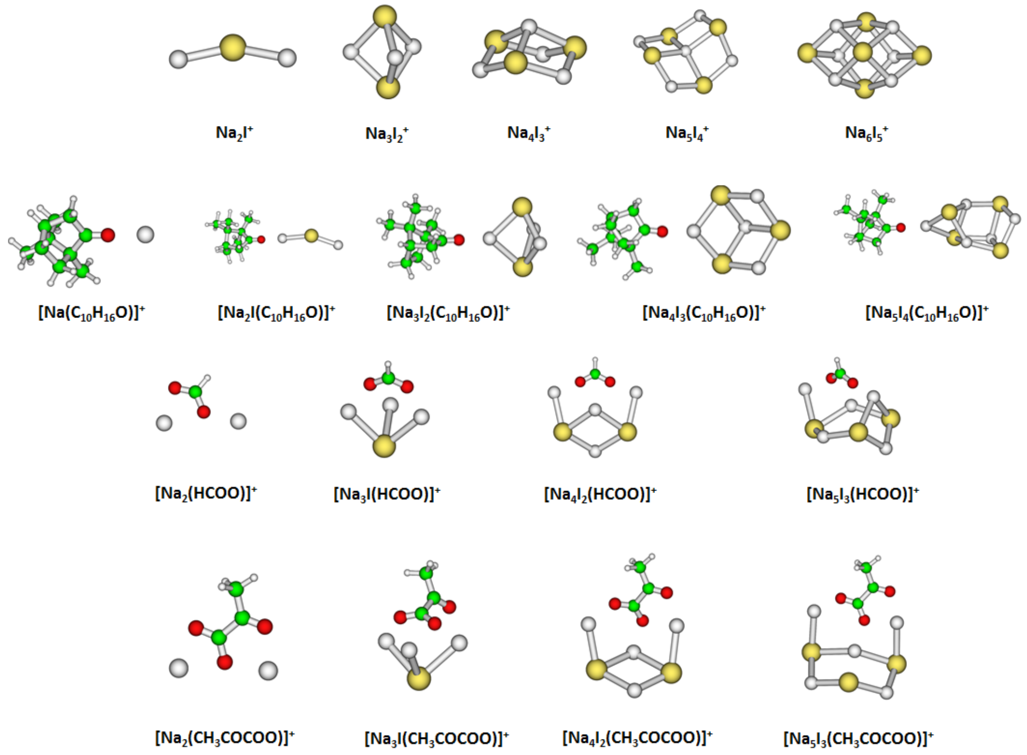

Figure S6: Structures of the most stable ions found at the B3LYP/def2TZVP level of theory. Relative energy is given in kJ/mol.

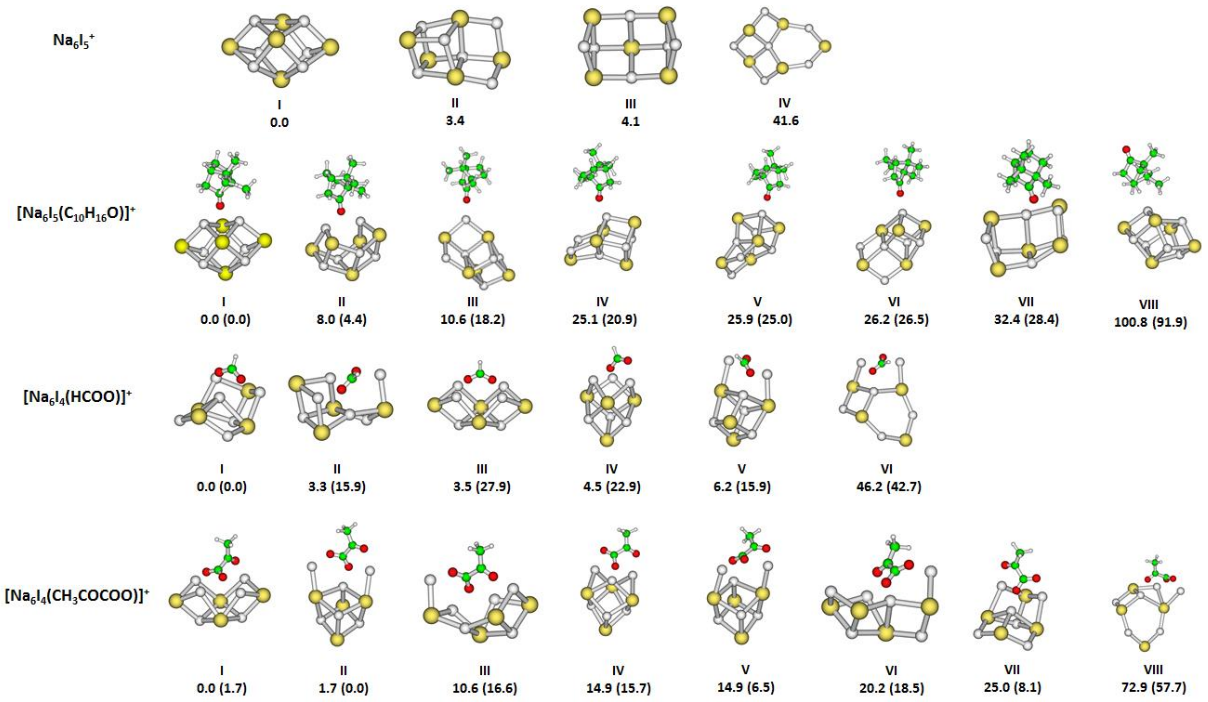

Figure S7: Isomers of $[Na_6I_5(C_{10}H_{16}O)]^+$, $[Na_6I_4(HCOO)]^+$, and $[Na_6I_4(CH_3COCOO)]^+$ clusters calculated at the B3LYP/def2TZVP level or MP2/def2TZVP,ECP(Na,I) level. Relative energy is given in kJ/mol, the MP2 values are given in parentheses.

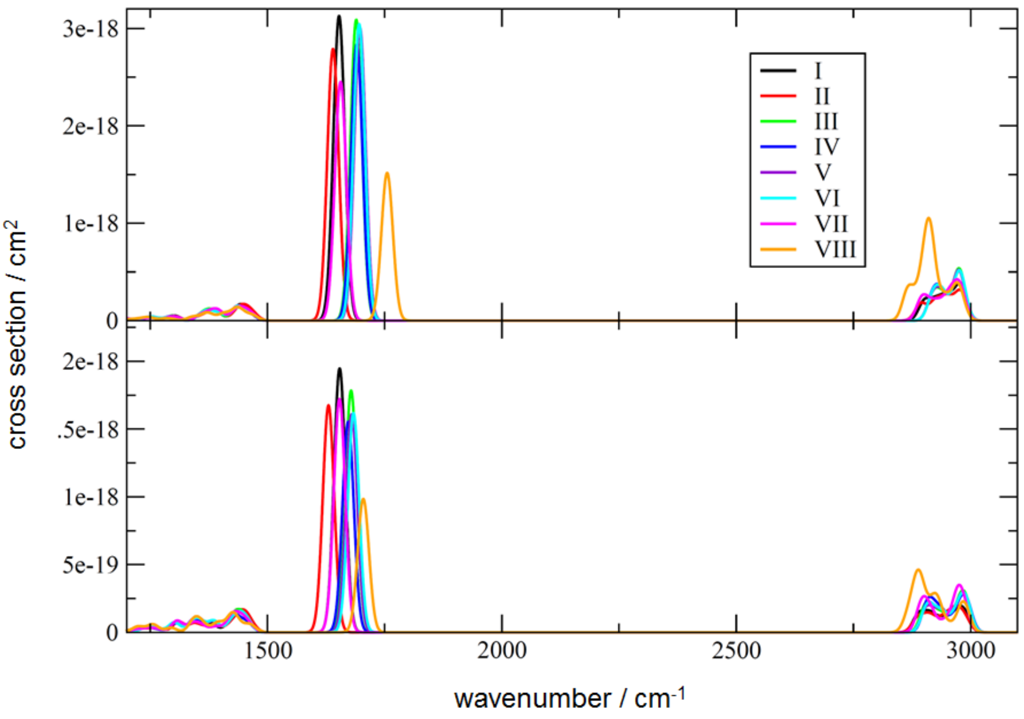

Figure S8: IR spectra of $[Na_6I_5(C_{10}H_{16}O)]^+$ isomers calculated at the B3LYP/def2TZVP (top) and MP2/def2TZVP,ECP(Na,I) (bottom) level of theory, scaled with a factor of 0.96 and 0.95, respectively. See Figure S7 for the respective structures.

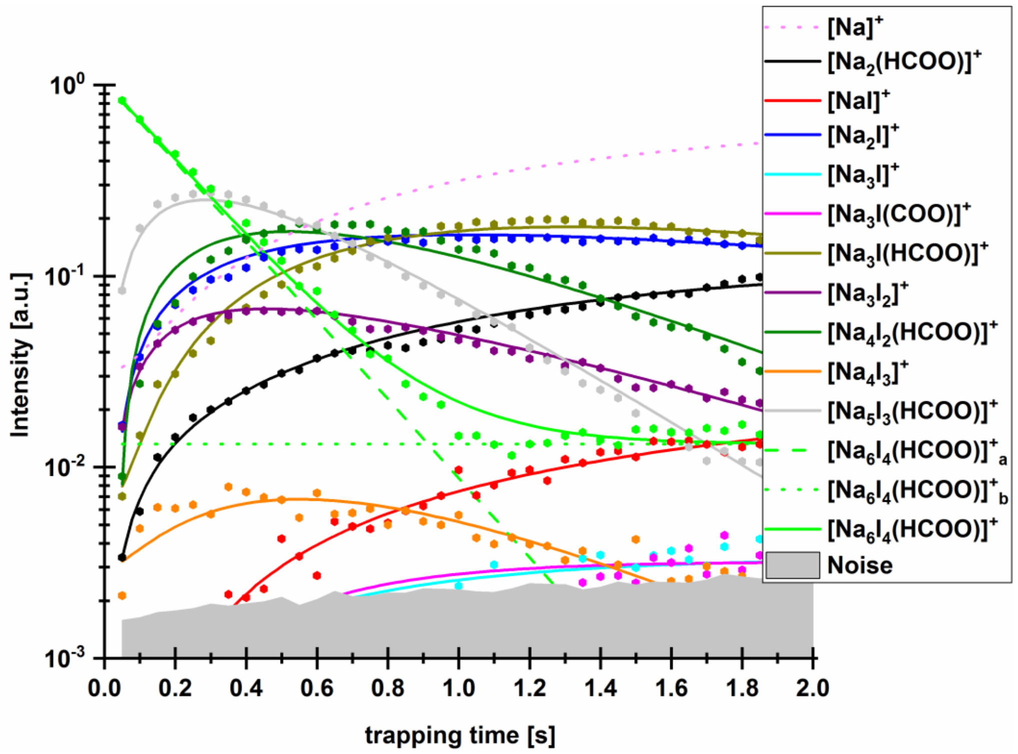

Figure S9: Kinetics of $[Na_6I_4(HCOO)]^+$ at 225 nm. The non-stoichiometric fragments $[NaI]^+$, $[Na_3I]^+$, $[Na_3I(COO)]^+$ are clearly secondary fragments. The dashed light green line represents the fraction of the precursor which fragments, while the dotted line represents the fraction of about 2.5% which does not fragment, most likely because a small part of the ion cloud that does not overlap with the laser beam. Na^+ lies outside mass window of the instrument and was fitted as a dark channel.

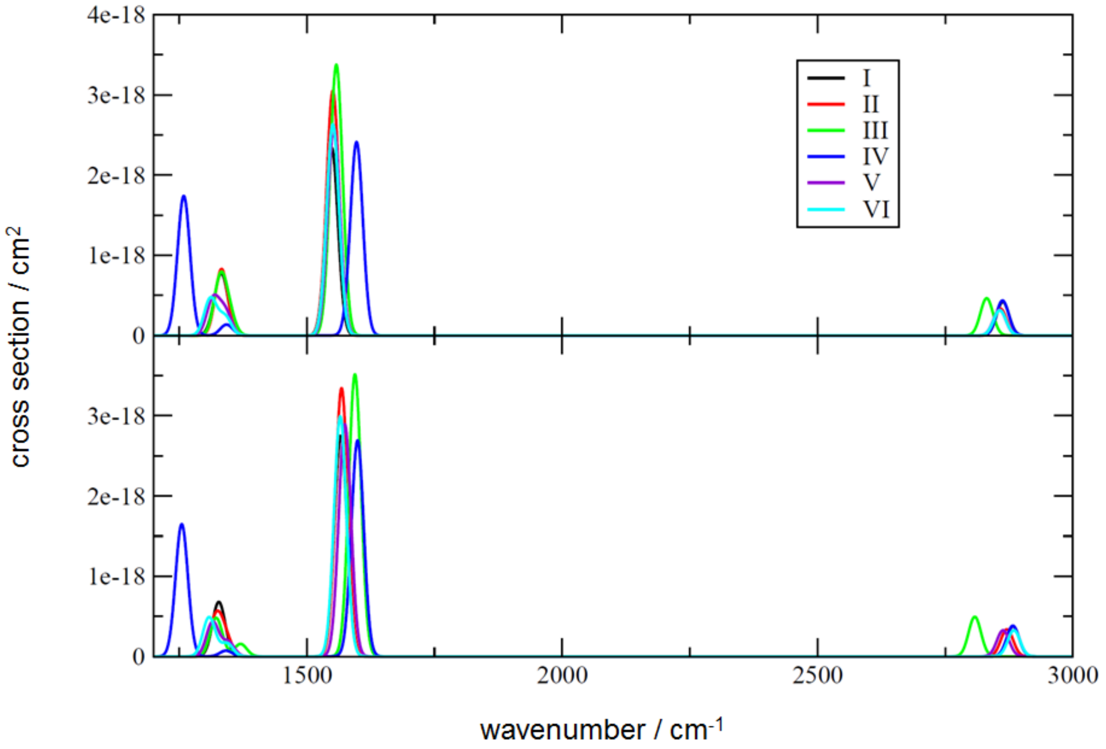

Figure S10: IR spectra of $[Na_6I_4(HCOO)]^+$ isomers calculated at the B3LYP/def2TZVP (top) and MP2/def2TZVP,ECP(Na,I) (bottom) level of theory, scaled with a factor of 0.96 and 0.95, respectively. See Figure S7 for the respective structures.

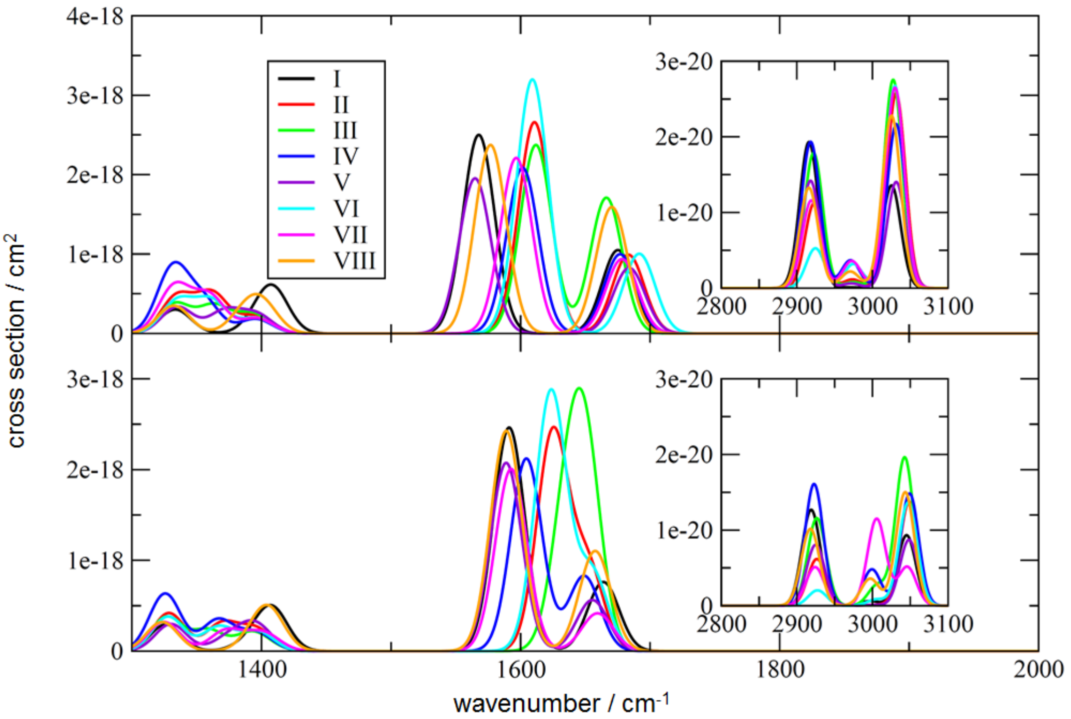

Figure S11: IR spectra of $[Na_6I_4(CH_3COCOO)]^+$ isomers calculated at the B3LYP/def2TZVP (top) and MP2/def2TZVP,ECP(Na,I) (bottom) level of theory, scaled with a factor of 0.96 and 0.95, respectively. See Figure S7 for the respective structures.

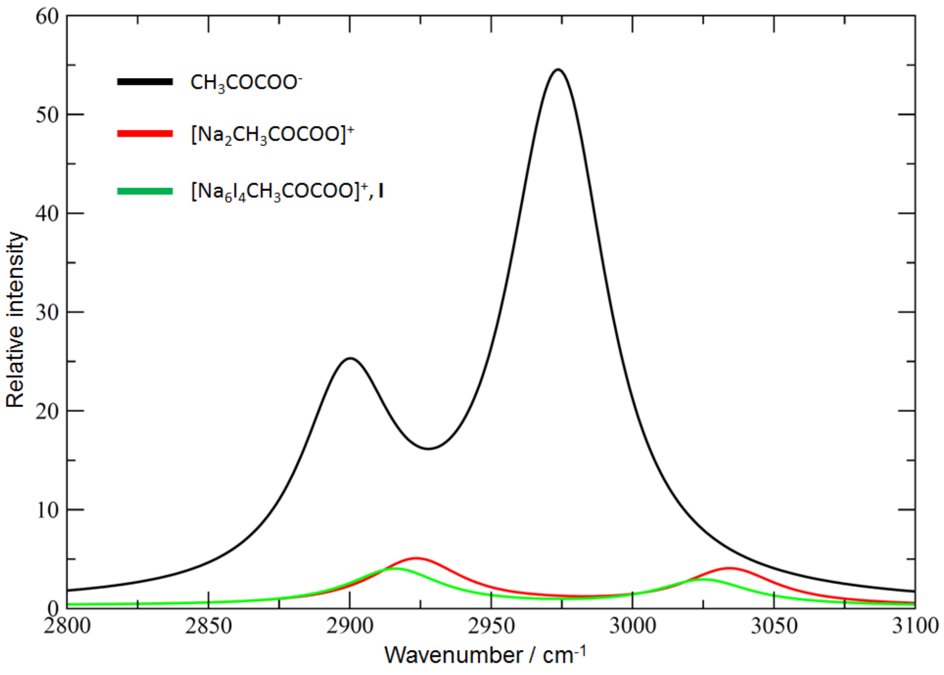

Figure S12: Spectra of several ions containing pyruvate in the C-H vibration region, calculated at the B3LYP/def2TZVP level of theory, scaled with a factor of 0.96. See Figures S6 and S7 for the respective structures.

Table S1: Reaction energies for various decomposition reactions of NaI clusters calculated at various levels of theory along with the def2TZVP basis set.

reaction	B3LYP	B3LYP+D2	MP2	CCSD(T)//MP2
$Na_2I^+ \rightarrow Na^+ + NaI$	1.69	1.78	1.70	1.70
$Na_3I_2^+ \rightarrow Na_2I^+ + NaI$	1.46	1.95	1.55	1.53
$Na_3I_2^+ \rightarrow Na^+ + Na_2I_2$	1.49	1.77	1.47	1.46
$Na_4I_3^+ \rightarrow Na_3I_2^+ + NaI$	1.70	1.98	1.88	1.86
$Na_4I_3^+ \rightarrow Na_2I^+ + Na_2I_2$	1.50	1.97	1.65	1.62
$Na_4I_3^+ \rightarrow Na^+ + Na_3I_3$	1.87	2.32	1.96	1.93

Table S2: Dependence of calculated excited state energies (in eV) and oscillator strengths (in parenthesis) on the used method and basis set.

molecule	basis	BHandHLYP	CAM-B3LYP	EOM-CCSD
$C_{10}H_{16}O$	def2SVP	4.55 (1.5e-4)	4.36 (1.5e-4)	4.47(1.3e-4)
	def2TZVP	4.55 (1.7e-4)	4.36 (1.7e-4)	-
	aug-cc-pVDZ	4.52 (1.6e-4)	4.32 (1.6e-4)	-
	aug-cc-pVTZ	4.54 (1.6e-4)	4.35 (1.6e-4)	-
$HCOO^-$	def2TZVP	6.38 (3.4e-3); 6.52 (0.0)	6.11 (3.2e-3); 6.21 (0.0)	6.32 (2.5e-3); 6.42 (0.0)
HCOOH	def2SVP	6.09 (1.6e-3)	5.91 (1.5e-3)	6.08 (1.5e-3)
	def2TZVP	6.08 (1.3e-3)	5.89 (1.2e-3)	6.00 (1.0e-3)
	aug-cc-pVDZ	6.01 (1.1e-3)	5.80 (9.7e-4)	5.94 (7.7e-4)
	aug-cc-pVTZ	6.03 (1.1e-3)	5.82 (9.4e-4)	5.93 (7.6e-4)
CH_3COCOO^-	def2TZVP	4.24 (2.4e-4)	3.97 (5.0e-4)	4.17 (3.3e-4)
$CH_3COCOOH$	def2SVP	3.79 (6.7e-6)	3.59 (1.2e-5)	3.83 (5.7e-6)
	def2TZVP	3.79 (1.5e-5)	3.58 (1.1e-5)	3.78 (2.3e-5)
	aug-cc-pVDZ	3.77 (4.4e-5)	3.56 (4.2e-5)	3.76 (7.5e-5)
	aug-cc-pVTZ	3.78 (4.2e-5)	3.57 (4.0e-5)	3.77 (6.8e-5)

Table S3: Benchmark of excited states in the $Na_4I_3^+$ ion without inclusion of the spin-orbit coupling, calculated using various methods and basis sets, excited state energies *E* (in eV) and oscillator strengths *f* are shown. The calculations were performed in the C_s symmetry subgroup as the full symmetry group of C_{3v} is not supported in the Molpro program, degenerate *A'*/*A''* states correspond to *E* within C_{3v}. The ECP10SDF(Na),ECP46MDF(I) basis set was used unless noted otherwise.

state	EOM-CCSD/ ECP10SDF(Na),ECP28MDF/VDZ(I)		EOM-CCSD		CAS(18,12)		MRCI(18,12)	
	E	*f*	*E*	*f*	*E*	*f*	*E*	*f*
2*A*'/1*A*''	5.70	0.17	5.43	0.20	5.83	0.24	5.54	0.21
3*A*'/2*A*''	5.78	0.05	5.54	0.02	5.91	0.00	5.64	0.01
3*A*''	5.93	0.00	5.69	0.00	6.03	0.00	5.79	0.00
4*A*'	6.04	0.37	5.84	0.41	6.19	0.48	5.95	0.44

Structure of atoms, molecules and ions (Cartesian coordinates, in Ångstrom) optimized at the B3LYP/def2TZVP level of theory along with the electronic energy including zero-point correction (in Hartrees)

Na+
E=-162.092539
Na 0.000000 0.000000 0.000000

I
E=-297.778401
I 0.000000 0.000000 0.000000

NaI
E=-460.185141
Na 0.000000 0.000000 -2.253165
I -0.000000 -0.000000 0.467638

Na2I2
E=-920.431305
I -0.000079 2.378999 0.000000
Na -0.000079 0.000000 -1.697220
I 0.000112 -2.378999 0.000000
Na -0.000079 0.000000 1.697220

Na3I3
E=-1380.665133
I -1.615966 -2.752555 0.000001
Na -2.484923 0.017904 -0.000001
I 3.192520 -0.023015 0.000004
Na 1.225838 -2.157187 -0.000014
I -1.576083 2.775569 0.000001
Na 1.256817 2.139287 -0.000015

Na4I4
E=-1840.915664
Na -0.700211 1.712917 -1.341123
I -0.917528 2.215803 1.655682
Na 1.715237 0.722133 1.327393
I 2.213594 0.890611 -1.673410
Na 0.719738 -1.736093 -1.300754
I -2.189309 -0.919934 -1.689464
I 0.893129 -2.186473 1.707142
Na -1.734214 -0.698991 1.314722

Na5I5
E=-2301.141110
Na 1.493726 0.987387 1.829964
I 0.187372 -1.692779 2.334715
I 0.075841 3.046666 0.012967
Na -1.814997 -1.577286 -0.006102
I 0.187911 -1.664184 -2.349865
Na 2.055084 -2.170903 -0.010317
I 3.819622 0.280547 0.002844
Na 1.499920 1.008572 -1.823601
I -4.366222 -0.081756 0.000824
Na -2.773712 2.289487 0.002905

NaI2
E=-758.003695
na -0.000000 -0.000000 2.129743
i -0.000000 1.697539 -0.221011
i -0.000000 -1.697539 -0.221011

Na2I3
E=-1218.237359
I -1.845008 1.650350 -0.000016
I 3.244143 0.000004 0.000009
Na 1.074122 1.914270 0.000044
I -1.844998 -1.650353 0.000023
Na 1.074133 -1.914274 -0.000119

Na3I4
E=-1678.470746
Na 2.011974 1.757693 -0.001781
I 1.888099 -0.807261 1.672208
Na -1.186181 0.109210 1.692555
I -0.751477 2.699899 -0.000660
Na -1.188417 0.108752 -1.692414
I -2.946695 -1.492987 0.001451
I 1.885334 -0.809694 -1.672658

Na2I+
E=-622.339763
Na 0.000000 2.783124 -0.434256
I -0.000000 0.000000 0.180257
Na -0.000000 -2.783124 -0.434256

Na3I2+
E=-1082.578527
I 2.121569 0.000000 0.000000
I -2.121569 0.000000 0.000000
Na 0.000000 0.000000 -2.153088
Na 0.000000 1.864629 1.076544
Na 0.000000 -1.864629 1.076544

Na4I2+
E=-1244.891138
I -2.309409 -0.370697 0.000084
Na -1.803102 2.657052 -0.000085
Na -0.000144 -0.870500 1.837079
I 2.309258 -0.371000 -0.000023
Na 1.804263 2.656849 0.000425
Na -0.000292 -0.869768 -1.837716

Na4I3+
E=-1542.826287
Na 0.321190 0.000000 2.887863
Na 0.321190 2.500963 -1.443931
Na 0.321190 -2.500963 -1.443931
I 1.302587 -2.364373 1.365072
I 1.302587 0.000000 -2.730143
I 1.302587 2.364373 1.365072
Na 2.728669 0.000000 0.000000

Na5I3+
E=-1705.132201
Na -1.763977 -3.612636 0.924554
I -2.778131 -1.004624 -0.281829
Na -2.676515 1.749823 0.808467
Na 1.763863 -3.612924 0.924447
I 2.777857 -1.004968 -0.281644
I 0.000112 2.798816 0.077021
Na 2.677382 1.749950 0.807251
Na 0.000027 -0.076837 -1.120907

Na5I4+
E=-2003.061704
Na 2.624890 -2.789333 -0.000471
I -0.089148 -2.936607 -1.155072
Na -2.789379 -2.624633 -0.000481
I 2.936562 -0.089454 1.155262
Na 2.789315 2.624698 0.001197
I 0.089527 2.936903 -1.154497
Na -2.624626 2.789197 -0.000584
I -2.937016 0.089213 1.154371
Na 0.000164 -0.000195 0.000026

Na6I4+
E=-2165.370386
Na 0.838300 -0.000045 1.987766
I -2.095718 -0.000032 2.336466
Na -1.908605 1.910226 -0.000452
I -2.094678 0.000035 -2.336774
Na -1.908606 -1.910221 -0.000509
Na 0.839308 0.000018 -1.987111
Na 4.424861 1.797538 0.000966
I 1.398853 2.331103 0.000020
Na 4.424861 -1.797439 0.000926
I 1.398876 -2.331121 -0.000042

Na6I5+, I
E=-2463.305603
Na 1.962855 1.206409 -1.778018
I 0.000091 -1.172713 -2.424729
Na 1.961559 -2.143745 -0.156000
I 4.018024 -0.000527 -0.000238
Na 1.963461 0.935544 1.934511
I 0.000081 2.687852 0.196845
I -0.000126 -1.513456 2.228340
Na -1.963600 0.935761 1.934430
I -4.018073 -0.000443 -0.000372
Na -1.962576 1.206348 -1.777952
Na -1.961686 -2.143751 -0.156230

Na6I5+, II
E=-2463.304326
Na -1.145509 0.000004 -2.359558
I 0.263104 -2.406358 -0.967860
I -3.795896 0.000003 -1.031019
Na -2.382243 -1.890732 0.860276
Na -2.382245 1.890728 0.860286
I -1.235570 -0.000004 2.907578
I 0.263105 2.406359 -0.967854
Na 1.114570 0.000000 1.064024
Na 3.240320 2.503350 -0.927919
I 4.155495 0.000001 0.356115
Na 3.240326 -2.503347 -0.927918

Na6I5+, III
E=-2463.304049
I -0.945962 0.072738 -0.518693
Na -0.271781 -0.134844 2.457070
Na 2.014657 0.712456 -0.619306
I 2.715693 0.537886 2.467906
Na 5.396517 -0.182636 1.164894
I 4.718219 0.032272 -1.808113
Na -1.487236 -2.768488 0.121184
I -0.230041 -3.174852 2.772332
Na 2.750975 -2.620921 2.738879
I 5.438164 -3.224670 1.472931
Na 5.424501 -2.821940 -1.461773

Na6I5+, IV
E=-2463.289758
Na 3.551738 -2.008711 0.930742
I -3.137900 -1.755239 -1.600573
Na -4.878517 0.000029 0.000147
I 0.612305 2.455651 -1.138510
Na 3.551756 2.008926 -0.930827
I -3.137907 1.755451 1.600717
Na -1.418362 -3.857788 -0.467430
I 5.382470 0.000173 0.000123

Na -1.418340 3.857825 0.467175
I 0.612353 -2.456098 1.138297
Na -0.984642 0.000015 -0.000064

Na3I+
E=-784.651671
Na 5.630563 0.248621 0.000955
I -0.862305 -0.232265 0.000154
Na 2.031842 0.124281 -0.001929
Na -3.507664 0.746194 0.000234

camphor
E=-465.857351
C -1.442658 0.066582 0.287430
C -0.863961 1.247856 1.073840
C 0.598565 1.261179 0.597838
C 0.584010 1.779927 -0.854854
C -0.070543 0.603394 -1.632304
C -0.294590 -0.502412 -0.550624
C 0.915648 -0.257949 0.418657
O -2.581537 -0.324769 0.316493
H -0.974854 1.061951 2.143166
H -1.412490 2.164377 0.849508
H 1.280122 1.796397 1.259715
H 1.589402 1.990198 -1.218526
H 0.013883 2.705746 -0.942283
H -1.008451 0.882193 -2.114739
C -0.545751 -1.890346 -1.098038
H 0.583183 0.217691 -2.415402
C 2.288814 -0.529596 -0.204732
C 0.831295 -1.073878 1.717048
H 3.078427 -0.196586 0.474000
H 2.432618 -1.599791 -0.367394
H 2.445176 -0.029187 -1.158589
H 1.585638 -0.732228 2.430159
H -0.139073 -1.018865 2.209930
H 1.029407 -2.128776 1.516834
H 0.281280 -2.221159 -1.729552
H -0.679409 -2.619154 -0.296865
H -1.457530 -1.903209 -1.697461

Na.camphor+
E=-628.008152
C 1.317986 1.134538 0.669374
C 1.378400 -0.410993 0.454010
C 1.088129 -1.235018 1.716749
C -0.155185 1.354499 1.059252
C -0.857847 0.329213 0.198214
C 0.188907 -0.416243 -0.583624
C 0.663138 0.682040 -1.608761
C 1.476176 1.693520 -0.757758
C -0.258434 -1.719876 -1.209183
C 2.707238 -0.906249 -0.124555
H 2.028554 1.528352 1.393878
H 1.855783 -1.053177 2.471107
H 0.122401 -1.021232 2.177348
H 1.107185 -2.302048 1.489601
H -0.360939 1.142497 2.110490
H -0.533317 2.359570 0.858581
O -2.073061 0.161855 0.138624
H 1.092341 2.710498 -0.837882
H 2.519855 1.724687 -1.064181
H 2.994029 -0.420018 -1.053959
H 2.669702 -1.980795 -0.310527
H 3.508252 -0.732661 0.597086
H 0.556840 -2.182191 -1.766013
H 1.274586 0.193236 -2.366535
H -1.078993 -1.556567 -1.911087
H -0.598086 -2.433779 -0.456529
H -0.178137 1.137673 -2.133334
Na -4.153329 -0.131043 0.073696

NaI.camphor
E=-926.070702
C -3.896115 -1.692854 -0.858995
C -3.843297 -1.168762 0.589644
C -2.392145 -1.357338 1.066639
C -1.663468 -0.287233 0.270515
C -2.698981 0.447396 -0.558543
C -3.079196 -0.625755 -1.637417
C -3.747784 1.175413 1.706723
C -5.253027 0.846478 -0.227724
C -2.252068 1.782364 -1.114044
H -1.989639 2.481749 -0.318565
H -1.371657 1.663511 -1.747852
H -3.038037 2.235261 -1.720121
H -3.670525 -0.142761 -2.415209
H -2.194329 -1.035195 -2.126585
O -0.464713 -0.082030 0.281932
H -1.974538 -2.344851 0.860279
H -2.255333 -1.169964 2.133029
H -3.463305 -2.690111 -0.940875
H -4.918798 -1.760444 -1.227138
H -4.588600 -1.601782 1.255917
H -2.795942 0.988603 2.205054
H -3.800323 2.247569 1.509546
H -4.542545 0.937560 2.417147
H -6.085508 0.635633 0.447818
H -5.237328 1.925282 -0.393291
H -5.476615 0.370893 -1.180463
Na 1.746025 0.043122 0.253992
I 4.500185 0.008421 -0.031618
C -3.939811 0.379401 0.407817

Na2I2.camphor
E=-1386.312417
C 3.672657 -1.214776 0.328496
C 4.256482 -1.863271 -0.974703
C 4.474995 0.136987 0.398866
C 2.321634 -0.708638 -0.141317
C 3.949508 1.105230 1.468907
C 4.598116 -0.657614 -1.893078
C 4.110872 0.547605 -1.063240
C 2.573941 0.478030 -1.055678
C 5.977197 -0.042471 0.636683
C 3.631983 -2.128941 1.533965
O 1.237759 -1.180725 0.146255
H 3.002453 -2.998053 1.336086
H 3.219209 -1.622364 2.407845
H 4.631151 -2.487705 1.786895
H 4.197663 0.741153 2.467689
H 2.870669 1.258231 1.440761
H 4.420292 2.084298 1.355447
H 6.165206 -0.396622 1.652100
H 6.451446 -0.741752 -0.048941
H 6.486134 0.918670 0.530393
H 4.509509 1.509591 -1.383681
H 4.090735 -0.711053 -2.856733
H 5.666036 -0.601579 -2.099755
H 5.141368 -2.442963 -0.711617
H 3.548442 -2.559181 -1.426841
H 2.128407 0.313567 -2.038913
H 2.089019 1.363170 -0.639360
Na -0.955065 -0.658519 0.130617
I -1.208902 2.366284 0.026137
I -3.751695 -1.688521 -0.006935
Na -3.864254 1.202531 -0.075390

Na3I3.camphor
E=-1846.543749
C -5.114681 0.627258 0.421956
C -5.871747 1.476126 -0.656162
C -5.717726 -0.806726 0.183050
C -3.743064 0.451816 -0.202743
C -4.987190 -1.913024 0.957793
C -6.116390 0.490153 -1.831625
C -5.409978 -0.792435 -1.347921
C -3.899691 -0.497985 -1.379695
C -7.208712 -0.917874 0.515511
C -5.113355 1.218029 1.815614
O -2.711744 0.981871 0.161389
H -4.628206 2.195481 1.818466
H -4.570614 0.584530 2.519090
H -6.131735 1.346278 2.186604
H -5.208394 -1.840121 2.024210
H -3.902522 -1.890588 0.853066
H -5.327702 -2.895569 0.623244
H -7.365760 -0.852005 1.593750
H -7.824328 -0.153489 0.045544
H -7.588408 -1.890566 0.193031
H -5.697048 -1.697179 -1.883022
H -5.695837 0.849842 -2.771336
H -7.179634 0.326398 -2.002481
H -6.804669 1.839648 -0.225043
H -5.297533 2.356489 -0.948269
H -3.554356 -0.029881 -2.303918
H -3.273403 -1.376909 -1.215848
Na -0.472192 0.808894 0.207466
I -0.049441 -2.172813 0.164943
I 1.482135 3.039272 0.079042
Na 2.829476 -2.295050 0.011388
Na 3.997501 1.643632 -0.056904
I 5.377431 -0.913094 -0.138972

Na4I4.camphor
E=-2306.794017
C 5.966421 -1.315228 1.407895
C 5.382341 -1.484235 -0.009076
C 3.868642 -1.233080 0.110150
C 3.820899 0.270478 0.332640
C 5.251541 0.777617 0.309115
C 5.831335 0.213186 1.651440
C 5.302711 -0.028503 -2.160771
C 7.405921 -0.107108 -0.860256
C 5.396339 2.272981 0.127959
H 4.980125 2.603753 -0.824866
H 4.864783 2.808649 0.916098
H 6.445439 2.571165 0.166338
H 6.794822 0.685142 1.844822
H 5.187578 0.455752 2.498018
O 2.823269 0.940364 0.513110
H 3.388388 -1.760513 0.937056
H 3.307831 -1.481318 -0.792906
H 5.415156 -1.904188 2.141396
H 7.005726 -1.637762 1.457483
H 5.639319 -2.424989 -0.495242
H 4.213487 -0.010735 -2.197002
H 5.653983 0.905521 -2.603296
H 5.638883 -0.840581 -2.809490
H 7.781202 -0.916092 -1.491965
H 7.699590 0.832644 -1.332142
H 7.923656 -0.171083 0.094969
Na 0.572379 0.780721 0.431008
I -1.708750 2.897750 0.327637
Na -2.470899 0.985532 -1.885624
I -0.025062 -0.768422 -2.282016
Na -1.735094 -2.245054 -0.265394
I -4.451043 -0.894702 -0.518300
Na -3.016892 0.567781 1.745339
I -0.721023 -1.295030 2.418826
C 5.880687 -0.202060 -0.748861

Na2I.camphor+
E=-1088.241612
C -4.461379 1.017877 -0.719399
C -4.399927 -0.492716 -0.330400
C -4.120312 -1.435613 -1.509268
C -3.032654 1.287077 -1.226258
C -2.207591 0.412662 -0.303248

C -3.159775 -0.303076 0.623826
C -3.650731 0.869500 1.549949
C -4.577203 1.725256 0.644837
C -2.587955 -1.497728 1.355808
C -5.658961 -1.002650 0.377485
H -5.240019 1.279285 -1.434173
H -4.938844 -1.392364 -2.230398
H -3.198269 -1.214245 -2.048585
H -4.049874 -2.468229 -1.163626
H -2.870925 0.972699 -2.259117
H -2.717545 2.330654 -1.158907
O -0.987435 0.330042 -0.302028
H -4.258341 2.766117 0.586841
H -5.601875 1.728746 1.011799
H -5.929283 -0.435787 1.265935
H -5.538399 -2.045662 0.675320
H -6.508048 -0.960526 -0.308254
H -3.336935 -1.944608 2.010257
H -4.184064 0.437122 2.395961
H -1.741021 -1.205472 1.979856
H -2.241600 -2.267877 0.664267
H -2.814191 1.434423 1.964055
Na 1.153431 0.129707 -0.255632
I 4.036732 -0.221108 -0.060068
Na 6.688465 0.650343 0.334599

Na3I2.camphor+
E=-1548.476494
C 4.003657 -0.154398 0.699315
C 4.547322 -1.628927 0.677051
C 2.981542 -0.176417 -0.412218
C 3.733273 -0.273551 -1.725713
C 5.196117 -0.324082 -1.248402
C 5.176811 0.625160 -0.009062
C 5.398666 -1.716102 -0.618207
C 4.828204 2.083979 -0.338403
C 6.486093 0.633535 0.786101
C 3.496348 0.318007 2.044477
O 1.766584 -0.142739 -0.287624
H 3.736612 -2.358402 0.708163
H 5.144630 -1.787474 1.574452
H 3.114770 1.339416 1.995557
H 4.291583 0.289859 2.790116
H 2.687978 -0.322802 2.402383
H 3.413026 -1.149974 -2.293306
H 3.504652 0.602183 -2.336217
H 5.921520 -0.063901 -2.017613
H 5.064867 -2.512074 -1.284143
H 6.446535 -1.909127 -0.395882
H 3.869814 2.210349 -0.843322
H 5.596197 2.520441 -0.980050
H 7.286545 1.053165 0.172831
H 6.394562 1.265296 1.671219
H 6.809570 -0.351276 1.115862
H 4.791728 2.682984 0.573041
Na -0.387696 -0.059926 -0.115636
I -2.742588 -2.109865 0.058439
I -2.588711 2.159134 -0.050590
Na -3.751973 0.018334 -1.786011
Na -3.563659 0.105117 1.892767

Na4I3.camphor+
E=-2008.720302
C 6.401377 -1.319934 0.265416
C 6.469727 0.119954 -0.334432
C 6.180606 0.187288 -1.840978
C 4.927109 -1.703402 0.041358
C 4.225399 -0.373880 0.250408
C 5.286094 0.658564 0.555519
C 5.753789 0.234485 1.994490
C 6.560468 -1.073946 1.778955
C 4.845999 2.101088 0.428484
C 7.805136 0.829117 -0.090137
H 7.111777 -2.029596 -0.156076
H 6.943094 -0.361880 -2.396959
H 5.210341 -0.220647 -2.126554
H 6.209262 1.221073 -2.188972
H 4.718860 -2.067088 -0.966566
H 4.546595 -2.456623 0.734595
O 3.020260 -0.186932 0.198477
H 6.169823 -1.906412 2.364746
H 7.604729 -0.951718 2.060629
H 8.091622 0.883087 0.957881
H 7.775864 1.848848 -0.477626
H 8.602817 0.305333 -0.621705
H 5.664071 2.778650 0.675312
H 6.367339 1.034798 2.407202
H 4.020617 2.316613 1.109350
H 4.506693 2.333416 -0.582393
H 4.907377 0.109939 2.671739
Na 0.865084 -0.066314 -0.072450
I -1.104302 -2.384139 -0.585377
Na -2.707116 0.022473 -1.527896
I -0.998218 2.339760 -0.548707
Na -3.512244 -2.448727 1.117090
I -5.011381 0.058655 0.554043
Na -3.401223 2.487571 1.155557

Na5I4.camphor+
E=-2468.957277
C -5.417148 -0.414625 -0.302708
C -7.351947 -0.368525 1.878546
C -6.402511 0.730384 -0.280515
C -6.884486 0.784011 -1.775001
C -7.628899 0.033415 0.422751
C -8.910283 0.872464 0.412294
C -5.869742 2.038117 0.264060
C -7.650515 -1.166606 -0.576752
C -7.775377 -0.475067 -1.949036
C -6.207480 -1.696056 -0.488160
H -6.035310 -2.351805 0.367442
O -4.202645 -0.334345 -0.205430
H -6.413491 -0.905402 2.021577
H -8.154614 -1.006891 2.253163
H -7.316869 0.514218 2.519045
H -5.530978 1.937638 1.296705
H -5.024217 2.390380 -0.330134
H -6.637409 2.812043 0.233638
H -8.814545 1.728563 1.082305
H -7.443936 1.707443 -1.922193
H -9.182490 1.251380 -0.570387
H -9.746946 0.271563 0.775544
H -6.043131 0.814051 -2.468984
H -8.806359 -0.207754 -2.173470
H -7.432556 -1.120713 -2.757813
H -8.411089 -1.919781 -0.376727
H -5.869563 -2.235610 -1.375570
Na -2.057509 -0.231559 0.089257
I 4.117191 2.408044 -0.176653
Na 3.477156 0.040627 -2.031470
I 0.404500 -0.123474 -1.857120
Na 1.253019 1.819897 0.521614
I -0.106791 -0.112071 2.418283
Na 1.451091 -1.924100 0.555862
I 4.366192 -2.206028 -0.128766
Na 5.483255 0.181263 1.217646

Na6I5.camphor+, I
E=-2929.202457
C 6.216138 -1.305707 1.523011
C 5.983857 -1.630158 0.034595
C 6.003431 -0.222453 -0.642564
C 4.856333 0.346103 0.279285
C 5.402576 0.001369 1.714136
C 3.798967 -0.709471 0.080762
C 4.504546 -2.037929 -0.093942
C 7.338517 0.514259 -0.500816
C 5.637711 -0.236138 -2.133871
O 2.582817 -0.539464 0.073172
C 4.399247 1.779406 0.107544
Na 0.638334 -1.896008 0.016818
I -0.139198 -0.074723 -2.532184
Na -2.661543 -1.680508 -1.892172
I -1.715303 -3.812117 -0.038147
Na -2.733093 -1.706754 1.809342
I -0.239899 -0.110825 2.561259
I -4.033182 0.503491 -0.052523
Na -2.124053 2.179437 1.843253
I -0.509587 3.912878 0.037123
Na 1.185972 1.386980 0.048425
Na -2.053103 2.201825 -1.850091
H 4.254094 -2.450664 -1.073504
H 4.174274 -2.761468 0.655412
H 6.677939 -2.354732 -0.387496
H 7.271302 -1.155941 1.742226
H 5.869437 -2.110767 2.170953
H 4.595085 -0.096361 2.440876
H 6.025427 0.830220 2.049201
H 8.116606 -0.032727 -1.037461
H 7.281087 1.508531 -0.947250
H 7.672859 0.632606 0.527367
H 6.385065 -0.795376 -2.699773
H 4.664766 -0.678201 -2.352645
H 5.625937 0.778977 -2.534060
H 5.236445 2.469420 0.213167
H 3.955505 1.953396 -0.875832
H 3.675985 2.057137 0.880294

Na6I5.camphor+, II
E=-2929.199430
O 2.364472 -0.343487 0.534847
C 3.571194 -0.577021 0.462032
C 4.697367 0.410049 0.618668
C 5.308397 -0.004331 2.009548
C 4.185082 -1.938712 0.211141
C 5.690673 -1.620634 0.258978
C 6.027079 -1.352526 1.738081
C 5.295134 -0.150232 -1.850690
C 5.751871 -0.199521 -0.385416
C 7.133957 0.455459 -0.308361
C 4.322658 1.873352 0.507707
H 3.838835 2.102050 -0.446059
H 3.671137 2.176840 1.333760
H 5.207317 2.506202 0.576914
H 4.284195 -0.524308 -2.019442
H 5.969219 -0.739046 -2.475275
H 5.323219 0.873944 -2.225757
H 7.536457 0.527014 0.699503
H 7.844352 -0.120673 -0.904689
H 7.108179 1.462546 -0.727775
H 6.313604 -2.372607 -0.221611
H 7.100131 -1.272757 1.898870
H 5.669641 -2.153048 2.385730
H 3.863748 -2.664128 0.963667
H 6.000752 0.777464 2.319978
H 4.543794 -0.070500 2.784952
H 3.852992 -2.308081 -0.762014
I -0.846007 2.715009 2.306443
Na -2.597778 0.286402 2.310056
I -1.518726 -2.502542 2.402583
Na 0.711484 -2.059313 0.456302
Na 1.295698 1.781337 0.419422
I -0.498962 -2.732469 -2.206237
Na -2.866810 -2.626811 -0.308118
I -3.759842 0.368732 -0.649033
Na -2.130542 3.041321 -0.413792
I 0.181611 2.492467 -2.292923
Na -1.459478 0.039631 -2.805126

Na6I5.camphor+, III
E=-2929.198420
C -8.119447 -0.477277 -0.162611
C -7.871981 -1.093008 1.251425
C -6.497476 -0.512182 1.627674
C -5.788728 -0.500422 0.286446
C -6.760982 -1.017441 -0.749120
C -6.844751 -2.543380 -0.384485
C -7.643722 -2.586903 0.946135
O -4.633234 -0.158628 0.092884
C -6.394512 -0.726502 -2.188657
C -9.364683 -1.024330 -0.867087
C -8.220065 1.055239 -0.171694
H -6.548465 0.506466 2.016600
H -5.940848 -1.104151 2.357267
H -8.652437 -0.892970 1.984008
H -8.583509 -3.123878 0.831586
H -7.092711 -3.080629 1.746932
H -5.854407 -2.994440 -0.306333
H -7.361527 -3.061582 -1.191834
H -10.260195 -0.725608 -0.317563
H -9.451736 -0.609672 -1.872884
H -9.382074 -2.108393 -0.955022
H -9.084541 1.381136 0.410290
H -7.343266 1.562533 0.232832
H -8.361042 1.421348 -1.189955
H -7.133783 -1.149768 -2.869724
H -6.331270 0.346126 -2.380429
H -5.426371 -1.165069 -2.437527
Na 0.838120 3.942694 -1.534610
I 2.987344 1.887711 -1.646068
Na 2.121708 -1.042675 -2.231491
I -1.140238 3.104635 0.458546
Na 1.188312 1.172104 1.052820
Na -2.545166 0.441605 0.009855
I -0.128508 -1.483993 -0.169991
I 4.385185 -2.570623 -1.052721
Na 4.946899 0.120171 0.209847
Na 2.498785 -2.516420 1.317723
I 3.356158 -0.006927 2.761270

Na6I5.camphor+, IV
E=-2929.192887
C 6.842177 0.603117 -0.561297
C 6.814555 -0.244737 0.749821
C 5.363288 -0.756480 0.782458
C 4.603856 0.427778 0.209841
C 5.619788 1.497189 -0.131063
C 6.072101 1.989476 1.289451
C 6.925297 0.822421 1.856916
O 3.394946 0.508288 0.071908
C 5.123500 2.587376 -1.056190
C 8.145309 1.381526 -0.766690
C 6.583834 -0.208505 -1.839215
H 5.196175 -1.632904 0.153637
H 4.996602 -1.008170 1.779822
H 7.563384 -1.033622 0.804235
H 7.957509 1.125773 2.023419
H 6.546046 0.453152 2.810085
H 5.217203 2.241258 1.918968
H 6.653529 2.903476 1.170464
H 8.972190 0.682977 -0.913562
H 8.086222 2.002591 -1.662245
H 8.409244 2.028898 0.066648
H 7.375639 -0.946006 -1.985619
H 5.633700 -0.743700 -1.842397
H 6.586808 0.445364 -2.712949
H 5.899827 3.333418 -1.231383
H 4.813635 2.184750 -2.022023
H 4.261711 3.097809 -0.622318
Na 1.333891 -0.294153 0.021149
I -0.709857 0.422248 -2.402915
Na -2.873357 0.404160 -0.016652
I -0.754927 0.379671 2.420958
I 0.883478 -3.311639 -0.012297
Na -1.407250 -2.700636 1.836618
I -3.750284 -2.454430 -0.047561
Na -1.969750 3.072164 2.518208
I -3.477672 3.489096 0.011606
Na -1.892731 3.129545 -2.457803
Na -1.372650 -2.667302 -1.893629

Na6I5.camphor+, V
E=-2929.192613
C 5.068282 -0.796205 -0.456988
O 3.890133 -0.486182 -0.446811
C 5.948600 -1.051459 0.748284
C 5.914992 -1.021396 -1.697022
C 7.265772 -1.430888 -1.085492
C 7.073606 -2.851659 -0.518499
C 6.138071 -2.608974 0.697315
C 7.335309 -0.545428 0.199868
C 7.364955 0.964582 -0.078302
C 8.523537 -0.869912 1.110087
H 7.376756 1.523653 0.858772
H 6.514640 1.327110 -0.657019
H 8.271705 1.231269 -0.625187
H 8.485939 -0.271231 2.021959
H 9.456942 -0.620906 0.600057
H 8.581320 -1.915503 1.404921
H 8.110480 -1.324277 -1.764696
H 6.632756 -3.521954 -1.256887
H 8.019241 -3.296196 -0.213043
H 5.181953 -3.127983 0.614910
H 6.589642 -2.932363 1.634861
H 5.463340 -1.776508 -2.343679
H 5.960422 -0.095633 -2.273402
H 5.294650 0.567333 2.035628
H 4.447901 -0.954029 2.297811
H 6.097682 -0.758652 2.881543
C 5.419740 -0.516638 2.062024
Na 1.855219 0.265405 -0.133961
I 0.941103 3.086553 -1.045186
Na -0.608117 2.763088 1.508955
I 0.246713 0.074065 2.506891
Na -2.062013 -0.847329 0.869306
I -3.477995 2.062392 0.213724
Na -1.442695 1.733244 -2.147213
I -0.856130 -1.282634 -2.055812
Na -3.142254 -3.144158 -1.945650
Na -5.753151 0.197866 0.558667
I -4.688417 -2.552565 0.506027

Na6I5.camphor+, VI
E=-2929.192467
C -5.519363 -1.681784 1.508148
C -7.001166 -1.898576 1.153581
C -7.031183 -2.967289 0.042773
C -6.426009 -2.211371 -1.171285
C -6.186334 -0.749480 -0.652042
C -5.014449 -0.929499 0.288989
C -7.359045 -0.585828 0.386882
C -8.755876 -0.596868 -0.241437
C -7.247604 0.687427 1.238331
C -5.993623 0.288848 -1.736054
O -3.869227 -0.556842 0.104261
Na -1.825980 0.238592 0.151564
I 0.705437 -1.421452 -1.861906
Na 1.283054 1.579067 -2.213428
I 3.498827 2.070150 -0.057716
Na 0.731946 2.871319 1.412210
I -0.015163 0.260613 2.667435
I -1.016756 2.999813 -1.024687
Na 2.153910 -0.781307 0.917065
I 4.744326 -2.518413 0.448740
Na 2.997832 -3.278702 -1.813742
Na 5.800569 0.226466 0.214341
H -5.368422 -1.068352 2.398204
H -4.952176 -2.602842 1.658011
H -7.634753 -2.118130 2.011830
H -8.044957 -3.306924 -0.162981
H -6.451143 -3.848923 0.317209
H -5.504233 -2.662523 -1.541648
H -7.114819 -2.170276 -2.014761
H -9.513159 -0.629521 0.545260
H -8.927024 0.315741 -0.815021
H -8.936199 -1.440991 -0.903442
H -7.971608 0.660002 2.055269
H -6.262267 0.842737 1.678781
H -7.472688 1.569609 0.636771
H -6.869601 0.340049 -2.383983
H -5.820788 1.281851 -1.318162
H -5.132636 0.042246 -2.359774

Na6I5.camphor+, VII
E=-2929.190119
C 4.898106 2.457286 1.012638
C 4.996637 2.483887 -0.545446
C 3.630818 1.916293 -0.973265
C 3.360868 0.897541 0.113194
C 4.535544 0.922755 1.061886
C 5.688028 0.295074 0.197283
C 6.037965 1.388620 -0.846442
O 2.369519 0.179030 0.195085
C 4.318247 0.254719 2.403770
C 6.206925 2.810817 1.725038
C 3.798898 3.362711 1.587898
H 2.826830 2.654412 -0.985674
H 3.637033 1.442043 -1.957862
H 5.223278 3.457490 -0.976828
H 7.055439 1.752790 -0.717778
H 5.957671 1.025960 -1.871005
H 5.381754 -0.647659 -0.259173
H 6.527261 0.066986 0.854032
H 6.474304 3.847049 1.507258
H 6.093279 2.730146 2.807700
H 7.050665 2.188776 1.435211
H 4.027418 4.409592 1.378748
H 2.804105 3.159011 1.189049
H 3.744121 3.255587 2.672865
H 5.163884 0.435868 3.067448
H 3.416785 0.614621 2.903268
H 4.248275 -0.833141 2.299422
Na -2.961316 -0.794509 1.419134
I -0.696223 -2.468211 2.490972
Na 1.997663 -1.854153 1.289070
I -2.523315 -1.466620 -1.591776
Na -0.817456 -3.712070 -0.335747
Na 0.546266 -0.230877 -1.464556
Na -3.236380 1.452011 -1.672985
I -4.122055 2.010812 1.212472
I 1.860285 -3.037197 -1.460969
I -0.455454 2.765767 -1.600294
Na -1.780786 3.742217 0.856162

Na6I5.camphor+, VIII
E=-2929.164060
C 7.070469 -0.596656 0.258229
O 8.255174 -0.731475 0.398469
C 6.010520 -1.708793 0.263516
C 6.566690 -3.099921 0.052769
C 5.277740 -1.487645 1.623080
C 4.467378 -0.176913 1.420061
C 4.857434 0.245303 -0.015651
C 6.317404 0.724840 0.060342
C 4.983028 -1.119378 -0.765636
C 5.540169 -1.006836 -2.191684
C 3.663499 -1.896839 -0.851624
H 3.161902 -2.022175 0.110561

H 2.983389 -1.406108 -1.560880
H 3.825435 -2.898849 -1.252181
H 4.884222 -0.395803 -2.817063
H 5.604759 -1.992802 -2.654979
H 6.536390 -0.569865 -2.234319
H 7.320519 -3.322771 0.808874
H 7.053298 -3.194076 -0.918912
H 5.784914 -3.859214 0.122424
H 5.983444 -1.421347 2.451073
H 4.628783 -2.338745 1.833946
H 4.724121 0.595341 2.144336
H 3.396519 -0.363169 1.550174
H 4.194227 0.979679 -0.483215
H 6.515508 1.411626 0.884155
H 6.662314 1.211111 -0.852556
Na 1.596355 0.223351 -0.167955
I 1.266448 3.217257 -0.045530
Na -1.028702 2.491456 -1.779942
Na -0.793596 2.328517 1.900020
I -3.438094 2.075521 0.211353
I -0.315123 -0.747931 2.231726
I -0.608308 -0.530807 -2.428976
Na -1.180636 -2.598082 -0.147721
Na -3.676144 -0.376758 -1.754632
Na -3.444235 -0.545033 1.961804
I -4.150783 -2.704266 0.039598

HCOO-
E=-189.267015
c -0.000000 0.000000 0.313397
h -0.000000 0.000000 1.455255
o 0.000000 1.134636 -0.208477

HCOOH
E=-189.814990
C 0.000000 0.420555 0.000000
O 1.157730 0.117321 0.000000
O -1.027415 -0.445694 0.000000
H -0.385443 1.448045 0.000000
H -0.657075 -1.344396 0.000000

Na2.HCOO+
E=-513.749607
C 0.238073 1.002569 0.000059
O 1.468768 1.043201 -0.000093
O -0.455743 -0.083943 -0.000031
Na -2.583337 -0.292830 -0.000013
Na 1.745852 -1.129469 0.000034
H -0.320293 1.955805 0.000400

Na3I.HCOO+
E=-973.996271
C -2.625836 -0.000002 -0.398150
O -2.195967 -1.114094 0.004719
O -2.195961 1.114091 0.004710
H -3.431375 -0.000003 -1.150483
Na -0.439290 -2.208461 -0.818912
I 1.429360 -0.000001 0.037855
Na -0.439283 2.208444 -0.818937
Na -1.069996 0.000026 1.770359

Na4I2.HCOO+
E=-1434.245633
C 0.002130 3.371906 0.174859
O 1.111125 2.823402 -0.044609
O -1.107454 2.824094 -0.043844
H 0.002551 4.395773 0.581503
I -2.406117 -0.900901 -0.074164
Na -3.045921 1.971157 0.398947
Na 0.000151 0.856828 -0.903210
Na -0.001368 -2.452287 0.735268
I 2.404709 -0.902820 -0.073201
Na 3.049860 1.968848 0.395114

Na2I.HCOO
E=-811.840679
C 3.314684 0.192214 0.000014
O 2.253987 0.892071 -0.000050
O 3.351541 -1.049733 -0.000003
Na 0.405899 1.929350 0.000021
Na 1.114691 -1.294889 0.000013
H 4.272380 0.744652 0.000072
I -1.617570 -0.143693 -0.000002

Na3I2.HCOO
E=-1272.074390
C 1.417063 3.805918 0.000595
O 2.642334 3.633697 0.001270
O 0.546819 2.871321 -0.001150
Na -1.457002 2.147318 -0.000754
I -3.022860 -0.288965 0.000163
Na -0.589020 -1.884633 -0.000050
I 2.278729 -1.556128 0.000029
Na 2.445432 1.380242 -0.000680
H 1.029844 4.842097 0.001631

Na4I3.HCOO
E=-1732.323643
C 1.058967 -0.000498 3.317800
O -0.010342 -0.000262 2.617742
O 2.216599 -0.000505 2.884360
H 0.916628 -0.000692 4.415161
Na -0.865489 1.675351 1.370301
I -2.909735 0.000524 -0.115269
Na -0.866250 -1.675361 1.370168
Na -0.388175 0.000206 -1.836770
I 1.182009 2.376597 -0.750772
I 1.181130 -2.376880 -0.750989
Na 2.488015 -0.000465 0.674826

Na5I3.HCOO+
E=-1894.478612
C 1.518446 -1.470703 2.702803
O 2.499333 -1.728361 1.955180
O 0.366865 -1.206981 2.286742
H 1.685879 -1.482043 3.792390
Na 0.993539 -1.673853 0.022572
Na -1.685880 -0.599758 1.887738
Na 4.291800 -0.851132 1.129621
I -1.924448 2.245095 0.542879
I 2.898943 0.605376 -1.074534
I -1.935226 -2.190753 -0.738189
Na -2.941308 0.411431 -1.596248
Na 0.904815 2.606399 -0.229400

Na6I4.HCOO+, I
E=-2354.718405
Na -1.580656 -0.000020 -1.996489
I -3.769191 -0.000003 0.035017
Na -2.083620 2.232995 1.279028
O -0.816235 -1.111589 2.705324
Na -2.083609 -2.232994 1.279051
I 0.063630 -2.444211 -1.027409
Na 3.016719 -2.438073 -0.752914
I 3.830532 0.000009 0.693708
Na 3.016716 2.438043 -0.752996
I 0.063620 2.444205 -1.027408
Na 0.731986 0.000013 1.255404
C -1.089458 0.000016 3.227734
O -0.816229 1.111619 2.705320
H -1.591790 0.000019 4.209372

Na6I4.HCOO+, II
E=-2354.717169
Na -0.580333 -2.207296 -0.352966
I -3.488949 -1.519769 -0.232488
Na -2.332888 0.574795 -2.075503
I -1.553593 2.835570 -0.249211
Na -2.443391 0.636144 1.626961
I 0.429365 -0.715439 2.191654
Na 3.287161 0.038947 2.333133
I 3.963814 0.377011 -0.534129
Na 3.315604 -2.223064 -1.801774
O 1.133118 -2.164084 -2.112689
Na 0.867878 1.099729 -0.528690
C 0.533794 -1.216369 -2.683606
O -0.222636 -0.400903 -2.094698
H 0.675286 -1.094398 -3.770816

Na6I4.HCOO+, III
E=-2354.717135
C 0.001164 0.687702 3.039159
O 1.123002 0.384747 2.559947
O -1.120002 0.383097 2.559363
Na -2.044666 -1.462010 1.575943
I -0.000909 -2.372470 -0.631451
Na 2.043476 -1.461780 1.575179
I 4.198771 -0.049737 0.049948
Na 1.939942 -0.211994 -1.848944
I 0.000835 2.168467 -1.040702
Na 2.250063 1.876530 1.189670
I -4.199177 -0.048008 0.050644
Na -2.247246 1.875450 1.189497
Na -1.942121 -0.213390 -1.850545
H 0.000482 1.292815 3.964512

Na6I4.HCOO+, IV
E=-2354.716689
O 3.497065 0.738898 0.000220
Na 2.183799 1.354737 -1.683652
Na 2.661848 -1.848408 -0.001324
Na 2.184080 1.352851 1.685041
I 0.010120 2.815373 0.001606
Na -1.681485 0.909832 1.876872
I -3.566473 -0.425107 -0.000042
Na -1.235403 -2.277740 -0.001214
Na -1.682048 0.912221 -1.875826
I 0.588494 -1.225036 2.336190
I 0.588060 -1.222229 -2.337639
C 4.631859 0.121174 -0.000083
O 4.761300 -1.101689 -0.000834
H 5.532516 0.757776 0.000378

Na6I4.HCOO+, V
E=-2354.716063
Na 0.281544 1.630974 1.673040
I 3.113592 1.366585 0.873074
Na 1.812738 1.045617 -1.834328
I 1.491377 -1.944579 -2.022828
Na 2.288298 -1.544401 0.866132
I -0.641248 -1.240355 2.285530
Na -3.574220 -1.103940 1.611474
O -4.657779 0.151094 -0.146960
Na -4.160386 2.225589 -0.711714
I -1.207675 2.010849 -1.020330
Na -1.234319 -1.577987 -0.913302
C -4.308078 -0.783894 -0.924968
O -3.466375 -1.664228 -0.634458
H -4.778955 -0.828431 -1.921810

Na6I4.HCOO+, VI
E=-2354.700799
Na 3.369835 -1.854337 0.567932
I 0.591141 -2.730721 1.187429
Na -1.340770 -3.603088 -0.877066
I -3.041768 -1.310268 -1.538642
Na -4.630094 0.350584 0.330549
O -3.041490 0.598150 2.007061
Na -2.745332 4.018338 0.131561

I -0.095916 2.692174 0.008133
Na 2.804222 2.453313 -0.622320
I 4.801520 0.391291 -0.517078
Na -1.203094 -0.197436 0.882739
C -3.287007 1.822525 2.078523
O -3.915109 2.477206 1.192684
H -2.941414 2.369670 2.971967

CH3COCOO-
E=-341.948742
C -0.011476 0.000000 0.000035
C -0.004540 0.000000 1.524140
C 1.398226 0.000000 2.158005
O 1.878660 -1.136400 2.346491
O -1.049528 0.000000 2.151026
O 1.878660 1.136400 2.346491
H 0.525145 0.880532 -0.361414
H -1.035870 0.000000 -0.378115
H 0.525145 -0.880532 -0.361414

CH3COCOOH
E=-342.481679
c 1.343219 1.383859 0.000000
c 0.000000 0.732978 0.000000
o -1.058776 1.319086 0.000000
c -0.033723 -0.817939 -0.000000
o 0.955178 -1.494741 -0.000000
o -1.275685 -1.303118 -0.000000
h 1.912160 1.054045 0.872026
h 1.234085 2.465335 0.000000

CH3COCOO
E=-341.811051
C -1.687942 0.866887 0.000027
C -0.708755 -0.261706 -0.000014
O -0.975331 -1.434079 -0.000040
C 0.786456 0.105484 -0.000005
O 1.234369 1.261691 -0.000045
O 1.667308 -0.795635 0.000053
H -1.522405 1.499068 0.876651
H -2.704818 0.481670 -0.000197
H -1.522100 1.499456 -0.876250

Na.CH3COCOO
E=-504.259558
C -2.153869 1.054994 -0.149727
C -1.336797 -0.200320 0.026665
C 0.186313 -0.022273 -0.001369
O 0.763788 -0.161270 -1.111256
O -1.821494 -1.295296 0.186621
O 0.751033 0.243695 1.092604
H -1.897881 1.770881 0.635187
H -3.215855 0.819164 -0.110456
H -1.907899 1.520159 -1.107723
Na 2.663740 0.054396 -0.001289

NaI.CH3COCOO
E=-802.059944
C 3.621205 -1.181462 0.837585
C 3.100075 -0.183637 -0.159806
C 1.587765 0.205785 -0.009335
O 0.819402 -0.754329 -0.232764
O 3.748351 0.305714 -1.043666
O 1.345537 1.387693 0.295687
H 3.429483 -0.827908 1.853666
H 4.687532 -1.338249 0.687917
H 3.075524 -2.119280 0.715272
Na -0.644367 2.205405 0.274657
I -1.910662 -0.387373 -0.046091

Na2I2.CH3COCOO
E=-1262.325637
C 5.321709 -0.099627 -0.674217
C 3.970522 0.351104 -0.227205
C 2.958616 -0.728049 0.253225
O 3.107554 -1.893751 -0.148822
O 2.010239 -0.303559 0.973582
O 3.610183 1.514079 -0.253247
H 5.209902 -0.812925 -1.493155
H 5.810813 -0.648202 0.134934
H 5.926724 0.750651 -0.979925
Na 0.849974 -2.217941 0.498976
I -1.958429 -1.510697 -0.131562
Na 1.379163 1.779757 0.534108
I -1.528303 1.772127 -0.051621

Na2I.CH3COCOO
E=-964.515093
C 4.300157 -0.238194 -0.709343
C 2.930671 -0.524720 -0.186720
C 2.057121 0.659318 0.294415
O 1.280132 0.408634 1.257600
O 2.440106 -1.641209 -0.166331
O 2.095636 1.709169 -0.371996
H 4.231496 0.503998 -1.506375
H 4.775758 -1.150075 -1.063253
H 4.900984 0.217843 0.082968
Na -0.170085 1.949942 0.298206
Na 0.229494 -1.531320 0.735297
I -2.204084 -0.139015 -0.208041

Na2.CH3COCOO+
E=-666.441515
C 0.010692 0.000000 0.001188
C 0.007372 0.000000 1.487928
O 1.019478 0.000000 2.165244
C -1.374820 0.000000 2.216331
O -2.397439 0.000000 1.516584
O -1.357525 0.000000 3.491321
Na -3.713049 0.000000 3.315148
Na 0.693228 0.000000 4.386259
H -0.538774 0.869781 -0.366756
H -0.538774 -0.869781 -0.366756
H 1.030080 0.000000 -0.376149

Na3I2.CH3COCOO
E=-1424.756485
C 0.954261 5.047947 -0.000021
C 1.304619 3.595559 0.000042
O 2.452596 3.195178 0.000105
C 0.128753 2.559385 -0.000014
O -1.021628 3.038565 -0.000061
O 0.463630 1.350037 0.000124
Na -0.179560 -0.928370 -0.000084
Na 2.731254 0.893532 -0.000081
Na -2.795841 1.738363 -0.000001
I 2.601981 -1.982505 0.000006
I -3.155296 -1.089796 0.000009
H 0.336546 5.275660 -0.871323
H 1.856790 5.654343 0.000527
H 0.335408 5.275583 0.870476

Na3I3.CH3COCOO
E=-1722.567142
C -2.372639 3.471102 0.000277
C -2.224382 4.957466 0.000485
O -3.455911 2.918444 0.000006
O -1.229734 1.361055 0.000220
C -1.066267 2.606667 0.000427
O 0.007279 3.237568 0.000737
H -3.201708 5.434118 0.000281
H -1.642945 5.268024 -0.870197
H -1.643431 5.267814 0.871569
Na -0.337482 -0.799887 0.000326
Na -3.434211 0.614142 -0.000195
I -2.938241 -2.225754 -0.000047
I 2.400443 -0.425168 -1.668000
Na 1.875991 2.073260 0.000531
I 2.400958 -0.426069 1.667599

Na3I.CH3COCOO+
E=-1126.674754
C -2.761492 0.334334 -0.338721
C -4.082220 -0.287981 -0.629481
C -1.758882 -0.379851 0.589185
O -1.316896 0.305437 1.542247
O -1.358341 -1.523734 0.252215
O -2.384156 1.395411 -0.809071
H -4.611401 0.271488 -1.397454
H -4.674781 -0.294274 0.291718
H -3.955447 -1.332355 -0.920015
Na -0.425111 2.127539 0.248692
I 1.957120 0.192367 -0.257666
Na 0.133196 -1.854584 -1.403335
Na 0.437820 -1.023522 2.070372

Na3.CH3COCOO+
E=-828.776878
C -0.942377 -2.133174 0.000647
C 0.242372 -1.187609 0.000204
C 0.045228 0.238349 0.000068
O 1.069912 1.039257 -0.000156
O 1.413295 -1.699633 -0.000026
O -1.165226 0.759909 0.000165
Na -2.973729 -0.351391 -0.000626
Na 2.933578 -0.177969 -0.000416
Na -0.224847 2.795218 0.000346
H -1.566066 -2.036186 -0.901896
H -1.566187 -2.035166 0.902976
H -0.567951 -3.154733 0.001206

Na4I.CH3COCOO+
E=-1288.985595
C 3.106117 -1.005888 -0.319200
C 4.576826 -0.829213 -0.477416
C 2.297962 0.087781 0.436556
O 2.678371 1.265326 0.314539
O 1.278324 -0.327603 1.051918
O 2.487797 -1.950125 -0.777821
H 5.042374 -0.743283 0.508734
H 5.005037 -1.665169 -1.024629
H 4.780336 0.112851 -0.990143
Na -0.455413 0.741439 1.911678
Na 0.266777 -2.046386 -0.174940
I -2.348812 -0.460857 -0.067150
Na -1.744554 2.234558 -1.278158
Na 1.771182 3.188947 -0.229827

Na4I2.CH3COCOO+
E=-1586.924846
C -2.524577 -1.050458 -0.266369
C -3.915563 -0.583807 0.266787
C -4.988470 -1.602597 0.443859
O -4.057960 0.593498 0.541021
O -1.822521 -0.151543 -0.800175
O -2.217214 -2.241599 -0.079616
H -5.155564 -2.131442 -0.498126
H -4.658980 -2.359085 1.159258
H -5.906925 -1.131405 0.784303
Na -0.398275 -3.392936 0.218649
Na 0.287371 0.061573 -1.505390
I 2.140467 -1.852186 -0.011050
Na 2.520655 0.824369 1.289482
I 0.648395 2.707266 -0.020870
Na -2.294751 1.972540 0.023697

Na4I3.CH3COCOO
E=-1885.001079
C 4.826224 -0.451193 -2.265943

C 3.640073 0.084975 -1.532657
C 2.339563 -0.757971 -1.527895
O 1.257605 -0.098438 -1.488584
O 2.460916 -1.988892 -1.459358
O 3.658139 1.121571 -0.895211
H 5.074912 -1.437995 -1.871462
H 5.670462 0.227888 -2.172721
H 4.571649 -0.598445 -3.319220
Na -0.851868 0.384482 -2.033968
Na 0.590411 -2.297892 -0.130991
I -2.371556 -1.868835 -0.734116
Na -1.735671 0.250297 1.400551
I -1.161197 2.778295 -0.231843
Na 1.614952 1.554231 0.221007
I 0.986309 -0.579697 2.400771

Na5I2.CH3COCOO+
E=-1749.271353
C -0.452951 1.883044 0.550975
C 0.576697 2.629547 -0.138368
C 0.236324 3.847234 -0.946579
O 1.819824 2.254059 -0.065339
O -1.697595 2.202592 0.439527
O -0.116591 0.851157 1.293179
H 0.363595 3.660950 -2.020649
H 0.919360 4.660509 -0.689433
H -0.784205 4.180042 -0.772159
Na 3.255943 1.290367 -1.378465
Na -3.061659 1.575164 -1.148698
Na -0.019538 -1.150933 0.032408
I -3.047264 -1.274424 -0.292053
I 3.001285 -1.374635 -0.087132
Na -2.290677 0.597438 1.989982
Na 2.091625 0.894983 1.726950

Na5I3.CH3COCOO+
E=-2047.161321
C 5.150692 2.280030 1.715218
C 4.317967 1.192058 1.127372
O 4.729666 0.061153 0.953266
C 2.847024 1.529777 0.704174
O 2.512159 2.726900 0.757224
O 2.146975 0.551020 0.338401
Na 0.829524 3.916829 0.094015
Na 0.023152 0.026586 -0.299986
Na 3.260466 -1.447644 0.058381
I -1.505667 2.659423 -1.189949
Na -3.632984 1.278429 0.310936
I -2.396681 -1.082208 1.543600
I 0.976874 -2.836615 -1.265689
Na -1.387167 -3.562977 0.299922
H 5.246765 3.096291 0.995504
H 4.644999 2.705534 2.584932
H 6.131021 1.899198 1.989836

Na6I3.CH3COCOO+
E=-2209.463630
C -3.135531 -2.014836 -1.397975
C -1.734083 -1.458097 -1.666920
O -0.774908 -2.085617 -1.167056
O -1.648536 -0.398577 -2.336382
O -3.739663 -1.605024 -0.419346
C -3.683251 -3.031541 -2.341410
H -3.000688 -3.883662 -2.397645
H -4.671345 -3.358447 -2.025661
H -3.730881 -2.603831 -3.347081
Na -3.560192 -0.064625 1.230882
Na -2.228180 1.740564 -1.956719
I -0.660245 -0.113616 2.541118
Na 0.802587 -0.414741 -2.081654
Na 0.377526 -2.498020 0.714306
I 3.004200 -1.475597 -0.272813
Na 2.025542 0.906985 1.171619
I 0.609781 2.635663 -0.996777
Na -1.464831 2.704808 1.303341

Na6I4.CH3COCOO+, I
E=-2507.403731
C 0.173002 -2.128458 -1.683393
C 1.220375 -3.215716 -1.415523
O 1.838199 -3.173520 -0.364735
C 1.424722 -4.269394 -2.451222
H 0.481113 -4.792721 -2.630309
H 1.705427 -3.800387 -3.397857
Na -1.736598 -0.162046 -2.068081
I -4.129225 -0.220154 -0.180619
Na -2.096695 -2.184835 0.777691
Na -2.243037 1.601094 1.158373
I -0.118207 -0.383220 2.525090
Na 1.640391 1.892880 1.254147
I -0.274708 2.528417 -1.061800
Na 1.927253 0.577914 -2.212300
I 3.924379 0.436413 0.037988
Na 2.429966 -1.821781 1.335122
O -0.940135 -2.273269 -1.131372
O 0.509578 -1.158687 -2.406817
H 2.191000 -4.974330 -2.137002

Na6I4.CH3COCOO+, II
E=-2507.403071
C 4.260186 0.349156 -0.263332
C 5.552227 -0.495360 -0.020524
O 5.424843 -1.671318 0.266337
C 6.874728 0.183134 -0.125621
H 6.928819 0.993473 0.604496
H 6.970249 0.655728 -1.106639
Na 3.280610 -2.488066 0.211929
I 0.310923 -2.461234 0.873097
Na -1.053986 -0.099159 2.352316
I 0.165257 2.455859 0.949020
Na 3.004692 3.192110 0.407372
I -3.758943 -0.113633 1.116074
Na -2.479120 1.837601 -0.794082
I -1.283917 0.036056 -2.891976
Na 1.051500 0.061276 -1.000204
Na -2.325117 -1.934345 -0.842804
O 3.226887 -0.311851 -0.544049
O 4.356699 1.582030 -0.122885
H 7.680338 -0.528762 0.034606

Na6I4.CH3COCOO+, III
E=-2507.399697
I 1.024766 -0.751855 2.681215
Na 2.534529 1.601096 1.483780
I 4.011441 0.380801 -0.800097
Na 1.193206 0.845095 -1.723575
I -0.044620 -2.042460 -1.622551
Na 2.479483 -1.978665 0.262823
Na -1.425549 -1.611336 1.154929
C -1.195771 2.694824 -0.843956
O -2.320128 3.075816 -0.472421
O -0.918261 1.787319 -1.667345
C 0.007218 3.381719 -0.141893
O 0.975256 2.692170 0.140395
C -0.098020 4.828353 0.199242
H 0.826259 5.191828 0.641758
H -0.935014 4.981075 0.883448
H -0.336808 5.401271 -0.701017
Na -4.223898 2.412784 0.337999
I -4.137851 -0.516666 0.237883
Na -2.283035 -0.009561 -2.099722

Na6I4.CH3COCOO+, IV
E=-2507.398050
O -2.940854 0.210908 -0.659676
Na -1.470145 0.281589 -2.296146
Na -2.016942 2.148214 0.610269
Na -2.304476 -1.810792 0.353748
I 0.136991 -2.253685 -1.633205
Na 1.545088 -1.977202 1.175848
I 3.915897 -0.257570 0.616223
Na 1.806056 1.696624 1.395796
Na 2.342267 0.001698 -1.892677
I -0.552591 -0.052142 2.443235
I 0.457644 2.489800 -1.347839
C -4.027320 0.565702 -0.085115
O -4.258803 1.685247 0.378488
C -5.058314 -0.563391 0.168985
O -4.620006 -1.672053 0.419215
C -6.506292 -0.219616 0.161145
H -6.691802 0.578600 0.882994
H -6.781779 0.187077 -0.816496
H -7.108822 -1.095437 0.388027

Na6I4.CH3COCOO+, V
E=-2507.398045
Na -1.101812 2.156715 0.908717
I -3.661973 0.680374 1.085599
Na -1.744049 -1.574194 1.717608
I -1.265563 -2.767275 -1.019527
Na -2.758103 -0.216557 -1.654700
I -0.270249 1.866135 -2.053589
Na 2.711845 2.030258 -1.807612
O 4.066669 1.257184 -0.060570
Na 3.924797 0.910170 2.246000
I 0.946740 0.176861 2.111767
Na 1.060134 -0.922747 -1.085311
C 4.003180 0.089328 -0.523241
O 3.242576 -0.281980 -1.449934
C 4.901015 -0.977866 0.160231
O 5.057324 -0.889498 1.363294
C 5.499477 -2.053276 -0.679228
H 6.132702 -1.603101 -1.449804
H 6.084658 -2.733089 -0.065135
H 4.712535 -2.591679 -1.210942

Na6I4.CH3COCOO+, VI
E=-2507.396031
Na 1.266740 2.102331 0.675313
I 3.964568 0.704374 0.252506
Na 3.411482 -0.667215 -2.292613
I 1.166477 -2.459863 -1.624456
Na 2.261662 -1.668923 1.119169
I -0.099653 -0.282109 2.491189
Na -2.950666 -1.079608 2.485908
I -4.045145 -0.970955 -0.252318
Na -4.173120 1.670057 -1.572517
O -0.524819 1.749692 -0.800469
Na -0.980307 -0.468212 -0.705792
C -1.195161 2.777580 -1.068386
O -2.357161 2.832282 -1.516819
C -0.495906 4.143031 -0.767636
O 0.460776 4.152274 -0.018705
C -1.053110 5.377343 -1.395238
H -1.111564 5.250833 -2.478864
H -2.079190 5.529654 -1.053644
H -0.439357 6.238385 -1.143012

Na6I4.CH3COCOO+, VII
E=-2507.394199
Na -0.832117 1.231283 -2.187534
I -3.507786 0.751990 -1.057916
Na -2.176341 1.657731 1.541983
O -1.152042 -0.932637 1.517656
Na -2.280137 -1.983294 -0.293489
I 0.446775 -1.625043 -2.096020
Na 3.354759 -1.997659 -1.632283
I 4.022875 -0.567581 0.874624
Na 3.500252 2.344075 0.768495

I 0.597320 2.668345 0.225489
Na 0.892612 -0.464128 0.849651
C -2.004970 -0.934059 2.460394
O -2.361164 0.045116 3.128549
C -2.756823 -2.271528 2.684842
O -3.010423 -2.945975 1.703104
C -3.182448 -2.625961 4.068284
H -2.306889 -2.676150 4.722019
H -3.812500 -1.828030 4.466495
H -3.712166 -3.575285 4.073491

Na6I4.CH3COCOO+, VIII
E=-2507.375954
Na 3.917868 1.111130 -1.170702
I 1.373986 2.652321 -1.328727
Na 0.235389 3.716646 1.197685
I -2.101576 2.074423 1.838655
Na -3.067224 -0.741030 1.661220
O -4.024222 -1.752200 -0.038664
Na -2.962785 -3.880041 -0.539701
I -0.506862 -2.341701 0.557647
Na 2.449736 -2.620493 0.848736
I 4.876441 -1.260405 0.146267
Na -1.123243 0.852198 -0.890829
C -4.076012 -1.677549 -1.300755
O -3.998657 -2.631415 -2.093801
C -4.112071 -0.287424 -1.945994
O -3.140170 0.435855 -1.794702
C -5.292143 0.081490 -2.778483
H -6.195979 0.051866 -2.162674
H -5.166469 1.071275 -3.211136
H -5.424342 -0.668470 -3.561576

Na2I2.CH3COCO
E=-1187.025974
C -1.659899 1.911778 -0.000248
O -0.655140 2.673917 -0.000279
C -3.048381 2.494231 -0.000281
H -2.958512 3.578582 -0.000811
H -3.618066 2.182722 0.877414
H -3.618440 2.181855 -0.877415
Na 1.352695 1.711131 -0.000186
Na 1.123735 -1.679401 -0.000063
I 3.613717 -0.219761 0.000201
I -3.102676 -0.891339 -0.000004
C -1.412338 0.493654 -0.000146
O -0.270759 -0.024153 -0.000070

Na3I3.CH3COCO
E=-1647.250750
C 4.935230 -1.150204 -1.446774
C 4.951692 -0.084314 -0.389363
C 6.141089 0.137066 0.301829
O 7.103034 0.351156 0.896215
O 3.970212 0.632612 -0.070580
Na 1.719044 0.670711 0.092702
I 1.121468 -2.281469 0.157198
Na -1.766105 -2.248309 0.080527
I -4.222325 -0.710041 -0.041907
Na -2.693286 1.763817 -0.034936
I -0.094444 3.008537 0.002147
H 4.140654 -1.863321 -1.214324
H 4.713095 -0.694288 -2.414209
H 5.886962 -1.678652 -1.516121

CH3CO
E=-153.205336
C 0.245880 -0.428642 -0.000003
C -1.166653 0.097606 -0.000003
O 1.258988 0.172638 0.000000
H -1.682189 -0.292677 0.878434
H -1.682311 -0.292926 -0.878255
H -1.182759 1.190715 -0.000144

NaI.CH3CO
E=-613.410351
C 2.590836 -1.576310 0.000914
C 3.259903 -0.242727 -0.002239
O 2.790411 0.849604 0.000007
H 2.932762 -2.127019 0.879647
H 2.932982 -2.132487 -0.874159
H 1.498812 -1.475069 0.000792
Na 0.659414 1.728379 0.000654
I -1.359355 -0.172834 -0.000105

Na2I2.CH3CO
E=-1073.652169
C -3.032924 2.975872 0.000141
O -1.868017 2.756140 0.000031
C -4.187878 2.027339 -0.000040
H -4.802867 2.236179 0.877214
H -3.847106 0.988114 -0.000245
H -4.802865 2.236523 -0.877214
Na -0.155908 1.184356 -0.000032
I 2.730543 0.531788 -0.000014
I -1.606505 -1.455048 -0.000016
Na 1.260246 -1.965848 0.000121

Na3I3.CH3CO
E=-1533.883296
Na -1.894508 2.072566 0.019713
Na -1.893359 -2.072896 0.019644
I -3.926949 -0.000731 0.086295
I 0.918711 -2.722703 -0.070293
Na 2.067594 0.000585 -0.126710
I 0.917356 2.723282 -0.070247
C 6.729388 -0.000172 0.590457
C 5.532779 0.000344 -0.306166
O 4.382657 0.000657 -0.028594
H 6.437050 -0.000027 1.643371
H 7.333784 -0.878373 0.357766
H 7.334633 0.877430 0.357733

Na3IO+
E=-859.916248
I 1.047702 0.000653 0.000433
Na -1.207126 1.680409 1.173662
O -1.974462 -0.001696 -0.000904
Na -1.202556 0.176240 -2.042563
Na -1.202366 -1.858562 0.867473

Na4I2O+
E=-1320.159255
Na -2.071823 2.357818 -0.631593
I -2.374700 -0.537473 0.056630
O 0.005675 2.475186 0.116788
Na -0.003247 -2.032349 -0.940897
I 2.371722 -0.542242 0.055797
Na 2.085431 2.356854 -0.624105
Na -0.000140 0.719806 1.569963

Na3I.CO2+
E=-973.348277
I 1.353533 0.000322 0.083001
Na -0.435893 2.208882 -0.962682
O -2.185912 1.127170 -0.030730
Na -0.433807 -2.209779 -0.961022
Na -1.157833 0.000724 1.840579
C -2.410177 -0.000536 -0.496942
O -2.185756 -1.128667 -0.032149

Na4I2.CO2+
E=-1433.596209
C -0.000041 3.237070 -0.221743
O -1.122161 2.800689 0.068838
O 1.122103 2.800688 0.068739
Na -3.060752 1.987557 -0.516887
I -2.392556 -0.858645 0.082602
Na 0.000006 0.838077 1.077213
Na 3.060699 1.987581 -0.517002
Na 0.000024 -2.378553 -0.818583
I 2.392575 -0.858614 0.082638

Na5I3.CO2+
E=-1893.831616
C -0.832461 -1.561491 2.376980
O -0.821009 -2.682753 1.827319
O -1.723992 -0.719792 2.475028
Na 1.231325 -3.231661 1.152358
Na -2.985866 -2.169551 0.947392
I 2.594089 -1.405090 -0.727759
I -2.780971 -0.091757 -1.131125
Na 0.162605 0.400628 -1.586418
Na -1.788506 1.334077 1.442876
I 0.705835 2.704163 0.396695
Na 3.185015 1.175740 0.663370

Na6I4.CO2+
E=-2354.079636
I -0.013608 -2.366208 -1.191370
Na 1.885352 2.087802 1.351532
Na 1.885324 -2.087500 1.351868
I 3.846052 0.000058 0.465250
Na 1.931383 -0.000140 -1.819470
I -0.013648 2.365940 -1.191668
Na -1.981656 -0.000168 -1.804346
I -3.876387 0.000028 0.510083
Na -1.844122 1.994599 1.368268
Na -1.843960 -1.994281 1.368429
O -0.000832 -1.139945 2.440162
C 0.451642 0.000284 2.597976
O -0.000793 1.140513 2.440014

Structure of atoms, molecules and ions (Cartesian coordinates, in Ångstrom) optimized at the MP2/def2TZVP,ECP(Na,I) level of theory along with the electronic energy including zero-point correction (in Hartrees)

Na6I4.HCOO+, I
E=-235.303854
Na 1.533730 0.000000 -2.190662
I 3.796345 -0.000000 -0.087022
Na 2.255370 -2.407251 1.244276
O 1.059287 1.116146 2.672340
Na 2.255371 2.407251 1.244276
I -0.132831 2.446277 -0.952380
Na -3.189347 2.675490 -0.662377
I -3.844536 0.000000 0.673497
Na -3.189347 -2.675490 -0.662375
I -0.132831 -2.446277 -0.952380
Na -0.696208 0.000001 1.449463
C 1.465881 0.000000 3.095018
O 1.059286 -1.116146 2.672340
H 2.225109 -0.000000 3.892949

Na6I4.HCOO+, II
na6i4.fa.iso2.mp2.ecp.com.log
E=-235.297786
Na -0.390668 -2.304947 0.014766
I -3.411598 -1.724445 0.034224
Na -2.413234 0.107881 -2.261546
I -1.797913 2.714639 -0.708605
Na -2.608736 0.835209 1.683229
I 0.471765 -0.202229 2.279900
Na 3.446462 0.560778 2.478649
I 3.966861 0.303613 -0.563196
Na 3.551396 -2.483006 -1.773858
O 1.272322 -2.393358 -1.812871
Na 0.870984 1.204003 -0.753785
C 0.675229 -1.491569 -2.458356
O -0.160914 -0.684853 -1.964694
H 0.896019 -1.397660 -3.534483

Na6I4.HCOO+, III
E=-235.293226
C 0.000034 -1.027377 2.707783
O -1.128465 -0.537168 2.449302
O 1.128615 -0.537333 2.449349
Na 2.092882 1.467556 1.765406
I -0.000065 2.362297 -0.526737
Na -2.093043 1.467602 1.765474
I -4.276119 0.072960 0.074498
Na -2.085034 0.357693 -2.019878
I 0.000057 -2.081244 -1.092567
Na -2.404170 -2.091673 1.130761
I 4.276104 0.073079 0.074483
Na 2.404379 -2.091738 1.130813
Na 2.084974 0.357709 -2.019849
H -0.000046 -2.014221 3.211216

Na6I4.HCOO+, IV
E=-235.295146
O -3.560347 0.656006 0.000031
Na -2.350575 1.382254 1.784513
Na -2.780992 -1.932043 0.000132
Na -2.350649 1.382190 -1.784542
I -0.071309 2.773310 -0.000065
Na 1.814105 1.023408 -2.050942
I 3.625501 -0.373031 -0.000041
Na 1.389573 -2.481183 0.000028
Na 1.814224 1.023541 2.050880
I -0.561816 -1.217639 -2.305045
I -0.561714 -1.217520 2.305125
C -4.723358 0.086354 0.000014
O -4.912520 -1.132249 0.000041
H -5.594550 0.760618 -0.000076

Na6I4.HCOO+, V
E=-235.297800
Na 0.234033 1.706978 1.854845
I 3.120593 1.385653 0.966162
Na 1.928940 1.234452 -1.931232
I 1.617026 -1.873284 -2.044895
Na 2.443542 -1.682755 0.975391
I -0.698750 -1.301749 2.205397
Na -3.775225 -1.342662 1.608224
O -4.697004 0.163149 -0.119733
Na -4.308083 2.327261 -0.722901
I -1.244737 1.975118 -0.995305
Na -1.269862 -1.650823 -1.098013
C -4.262012 -0.665351 -0.972812
O -3.521535 -1.644360 -0.711329
H -4.555457 -0.519230 -2.026141

Na6I4.HCOO+, VI
E=-235.287576
Na 3.650403 -2.294494 0.590323
I 0.627516 -2.779133 0.611102
Na -1.810413 -3.786718 -0.952339
I -3.609634 -1.350053 -0.940072
Na -4.728771 0.753678 0.967274
O -2.687578 1.041769 2.141426
Na -2.572121 4.265114 -0.213490
I 0.046675 2.688438 -0.412885
Na 3.082786 2.468855 -0.762829
I 4.964817 0.262913 -0.149511
Na -1.106700 -0.095566 0.841793
C -2.793338 2.286441 2.035851
O -3.602436 2.880088 1.256292
H -2.143641 2.912151 2.667542

Na6I4.CH3COCOO+, I
E=-387.645146
C -0.356019 1.821002 -1.869235
C -1.469403 2.839740 -1.618532
O -1.818942 3.067225 -0.467620
C -2.077395 3.498293 -2.807254
H -1.292818 3.979724 -3.396889
H -2.526704 2.736713 -3.447543
Na 1.736132 0.020020 -2.278676
I 4.136557 0.407331 -0.307221
Na 1.967498 2.419837 0.563616
Na 2.520259 -1.567000 1.339918
I 0.174641 0.527910 2.530789
Na -1.669683 -1.961213 1.545341
I 0.340320 -2.594003 -0.814287
Na -2.015616 -0.975519 -2.345650
I -3.843426 -0.362669 0.079988
Na -2.460642 2.035244 1.479608
O 0.750600 2.041175 -1.323413
O -0.634855 0.835668 -2.603927
H -2.824270 4.227602 -2.502746

Na6I4.CH3COCOO+, II
E=-387.645798
C -4.301820 0.294913 0.319285
C -5.603931 -0.489056 0.017974
O -5.524096 -1.674278 -0.279185
C -6.895634 0.248135 0.080257
H -6.890550 1.047860 -0.662101
H -6.994975 0.732738 1.053581
Na -3.388019 -2.629951 -0.168838
I -0.332527 -2.442658 -0.816520
Na 1.006978 -0.106392 -2.560521
I -0.248465 2.415489 -0.912065
Na -3.153837 3.294536 -0.411934
I 3.770830 -0.095406 -1.212780
Na 2.619999 2.030243 0.759904
I 1.432500 0.057777 2.880911
Na -1.133059 0.064275 1.171711
Na 2.495490 -2.096120 0.845836
O -3.303300 -0.407805 0.632790
O -4.358477 1.536797 0.197300
H -7.726149 -0.430476 -0.097475

Na6I4.CH3COCOO+, III
E=-387.639467
I 1.104625 -0.677656 2.658606
Na 2.558552 1.830989 1.533242
I 4.048749 0.571677 -0.836582
Na 1.165590 0.923349 -1.891950
I 0.051678 -2.087946 -1.544403
Na 2.746671 -2.040397 0.240565
Na -1.455737 -1.839311 1.347656
C -1.408405 2.546021 -0.952346
O -2.556584 2.951944 -0.682750
O -1.062188 1.674757 -1.792673
C -0.273652 3.166639 -0.099324
O 0.786676 2.559665 0.015791
C -0.548497 4.454525 0.595407
H 0.361569 4.857327 1.034285
H -1.293265 4.284733 1.375814
H -0.987140 5.169417 -0.101667
Na -4.562374 2.444572 0.143117
I -4.092638 -0.541560 0.288518
Na -2.361705 -0.212364 -2.272554

Na6I4.CH3COCOO+, IV
E=-387.639806
O -3.016797 0.189486 -0.614584
Na -1.654842 0.318243 -2.406565
Na -2.128632 2.197760 0.695899
Na -2.386253 -1.886062 0.391291
I 0.133771 -2.195292 -1.637442
Na 1.691018 -2.174789 1.245846
I 3.984180 -0.242357 0.571713
Na 1.946343 1.844674 1.532363
Na 2.489495 0.041971 -2.097162
I -0.511045 -0.089460 2.424184
I 0.417775 2.468633 -1.301846
C -4.112743 0.537470 -0.049394
O -4.372513 1.660137 0.407534
C -5.146196 -0.580399 0.178575
O -4.733419 -1.714653 0.388998
C -6.583885 -0.203938 0.197758
H -6.747280 0.524980 0.993892
H -6.845132 0.299416 -0.735952
H -7.205258 -1.083481 0.346174

Na6I4.CH3COCOO+, V
E=-387.643306
Na 1.031313 -2.323440 1.043253
I 3.631471 -0.748436 1.196173
Na 1.794605 1.703464 1.869732
I 1.436316 2.721811 -1.071430

Na 2.977327 0.074191 -1.763220
I 0.222282 -1.909622 -2.006172
Na -2.890126 -2.038295 -1.953047
O -4.144662 -1.192785 -0.074374
Na -4.102386 -0.937638 2.294509
I -1.070782 -0.088447 2.005867
Na -1.083228 1.021366 -1.203439
C -3.992443 -0.025301 -0.520964
O -3.255717 0.301467 -1.489515
C -4.770540 1.106523 0.185712
O -5.046915 0.978842 1.370024
C -5.136761 2.305800 -0.616870
H -5.678450 1.997852 -1.513776
H -5.738963 2.986596 -0.020362
H -4.230577 2.805929 -0.963110

Na6I4.CH3COCOO+, VI
E=-387.638758
Na 1.085983 2.055733 1.144828
I 3.910828 0.854922 0.463014
Na 3.707503 -0.156019 -2.412906
I 1.447558 -2.169069 -1.922956
Na 2.449886 -1.877274 1.085145
I -0.177096 -0.751435 2.410700
Na -3.142700 -1.538507 2.497839
I -4.099276 -0.892722 -0.331376
Na -4.254514 1.769227 -1.799590
O -0.533175 1.742385 -0.587759
Na -0.979028 -0.497884 -0.865704
C -1.232672 2.750242 -0.868320
O -2.319636 2.783916 -1.487552
C -0.691009 4.112511 -0.362949
O 0.162505 4.126507 0.512664
C -1.261114 5.355020 -0.957597
H -1.180531 5.315912 -2.045249
H -2.326774 5.408640 -0.728975
H -0.746630 6.228427 -0.564403

Na6I4.CH3COCOO+, VII
E=-387.642723
Na 0.785683 0.116189 -2.476508
I 3.414048 -0.181122 -0.918247
Na 2.143669 -2.656767 0.426713
O 0.993672 0.799276 1.987720
Na 2.200529 2.384326 0.755097
I -0.428790 2.686343 -1.155378
Na -3.217839 2.799504 0.030116
I -3.921936 0.018052 1.140041
Na -3.766082 -2.427255 -0.706490
I -0.698515 -2.453178 -1.125913
Na -0.811815 -0.573230 1.607252
C 1.786263 -0.140255 2.248238
O 1.504188 -1.369704 2.203672
C 3.215410 0.277992 2.671614
O 3.575887 1.424769 2.460927
C 4.082124 -0.739975 3.331897
H 3.520488 -1.306682 4.074127
H 4.429882 -1.446351 2.573977
H 4.946570 -0.253768 3.778203

Na6I4.CH3COCOO+, VIII
E=-387.623810
Na -4.354293 1.181159 1.179681
I -1.651394 2.625470 1.118619
Na -0.015792 4.238920 -0.933837
I 2.138655 2.242764 -1.656079
Na 3.407113 -0.524443 -1.590799
O 4.141268 -1.679979 0.187882
Na 3.206738 -3.932635 0.484471
I 0.695593 -2.352603 -0.701504
Na -2.320196 -2.842822 -1.158241
I -4.723564 -1.471607 -0.111479
Na 0.816814 0.631262 0.856125
C 4.009886 -1.643082 1.449253
O 3.907761 -2.622715 2.215240
C 3.862869 -0.285795 2.124088
O 2.863295 0.383048 1.883840
C 4.908148 0.121884 3.101765
H 5.881824 0.136146 2.605468
H 4.683226 1.098801 3.523339
H 4.964337 -0.634988 3.886927

Na6I5.camphor+, I
E=-522.365697
C 6.273695 -1.719152 1.007241
C 5.584604 -1.724250 -0.365334
C 5.517541 -0.218904 -0.726021
C 4.770119 0.178818 0.578422
C 5.680940 -0.454661 1.677443
C 3.629186 -0.789748 0.555290
C 4.112896 -2.059624 -0.100714
C 6.886526 0.432217 -0.869276
C 4.704373 0.085866 -1.981464
O 2.502668 -0.603271 1.016550
C 4.400031 1.621155 0.807410
Na 0.587419 -2.036550 0.843098
I 0.653782 -0.258583 -1.999959
Na -2.061162 -1.899317 -2.348026
I -1.822863 -3.826094 0.029413
Na -3.433135 -1.631424 1.457082
I -1.054444 0.078074 2.708532
I -3.736560 0.582438 -0.960375
Na -2.644680 2.533512 1.454174
I -0.326314 3.973924 0.038609
Na 1.265377 1.453235 0.905154
Na -1.252137 2.254005 -2.327974
H 3.534398 -2.209498 -1.018170
H 3.959106 -2.929880 0.544642
H 6.058525 -2.355639 -1.118412
H 7.357065 -1.651212 0.913487
H 6.056001 -2.629111 1.569486
H 5.121902 -0.670188 2.591775
H 6.451590 0.273316 1.938996
H 7.400591 0.009622 -1.736706
H 6.783635 1.505576 -1.045297
H 7.532674 0.295392 -0.003170
H 5.130889 -0.431055 -2.845116
H 3.652887 -0.194943 -1.911487
H 4.732094 1.156855 -2.198086
H 5.290945 2.250145 0.841321
H 3.767541 2.005038 -0.000008
H 3.890018 1.746162 1.768126

Na6I5.camphor+, II
E=-522.364009
O 2.039773 -0.366065 1.606372
C 3.194674 -0.646524 1.263803
C 4.362819 0.279927 1.181501
C 5.288180 -0.241966 2.327246
C 3.665005 -2.014584 0.827833
C 5.129284 -1.742807 0.463568
C 5.875095 -1.569498 1.791641
C 4.206415 -0.154457 -1.345791
C 5.060939 -0.296586 -0.088756
C 6.423242 0.327033 -0.357574
C 4.064427 1.755129 1.240203
H 3.391278 2.063895 0.437016
H 3.644093 2.029377 2.215067
H 4.981898 2.335565 1.130790
H 3.216045 -0.605115 -1.261024
H 4.706619 -0.637322 -2.189395
H 4.067565 0.899063 -1.599346
H 7.066266 0.372967 0.520822
H 6.945193 -0.253978 -1.122496
H 6.308173 1.342563 -0.743489
H 5.560294 -2.476631 -0.219087
H 6.951989 -1.497773 1.641550
H 5.698585 -2.407682 2.468037
H 3.543641 -2.750330 1.630903
H 6.059352 0.510166 2.505272
H 4.739393 -0.359708 3.265792
H 3.053824 -2.330966 -0.022376
I -1.850791 2.385643 2.293009
Na -3.727466 0.082757 1.469942
I -2.566304 -2.753682 1.657737
Na 0.422060 -2.146850 1.332162
Na 1.095216 1.855659 1.581272
I 0.674895 -2.395164 -1.801198
Na -2.456501 -2.717800 -1.456352
I -3.096204 0.445180 -1.806980
Na -1.962158 3.276042 -0.709457
I 1.053763 2.782272 -1.418160
Na -0.033596 0.369754 -2.974359

Na6I5.camphor+, III
E=-522.358784
C -7.444050 -0.031124 0.586355
C -7.957117 -1.475980 0.816753
C -6.653562 -2.277735 0.908378
C -5.767591 -1.527153 -0.063411
C -6.582443 -0.411164 -0.652989
C -7.611647 -1.168177 -1.542694
C -8.585521 -1.832037 -0.537832
O -4.594969 -1.782724 -0.306889
C -5.788844 0.658392 -1.356352
C -8.549294 0.968913 0.274753
C -6.609035 0.526250 1.736868
H -6.193386 -2.257954 1.899194
H -6.749694 -3.326730 0.614883
H -8.619684 -1.596357 1.675739
H -9.593952 -1.429431 -0.632643
H -8.655052 -2.912528 -0.675733
H -7.118034 -1.887787 -2.201506
H -8.113657 -0.441786 -2.185358
H -9.213944 1.061994 1.138082
H -8.126466 1.957598 0.080943
H -9.159107 0.692335 -0.584427
H -7.222716 0.622719 2.636323
H -5.743530 -0.089330 1.991028
H -6.232460 1.520890 1.486565
H -6.444146 1.460667 -1.701337
H -5.029090 1.092648 -0.703608
H -5.286238 0.246561 -2.236634
Na -0.228637 4.026242 -0.854650
I 2.462105 2.500820 -0.915549
Na 2.735835 -0.276319 -2.538329
I -2.080171 1.892404 0.316410
Na 0.667231 0.623133 1.258138
Na -2.525535 -1.071395 -0.752043
I 0.510823 -1.878678 -0.891795
I 5.242022 -1.358510 -1.142529
Na 4.909504 1.054883 0.829018
Na 3.221179 -2.730839 0.829595
I 3.053527 -0.380858 2.904135

Na6I5.camphor+, IV
E=-522.357727
C -6.383535 -0.583836 -0.289375
C -6.178874 -0.602449 1.246323
C -4.652160 -0.646025 1.369598
C -4.274153 -1.494245 0.172678
C -5.541717 -1.869655 -0.536650
C -6.191961 -2.875599 0.459583
C -6.682675 -1.998667 1.638581
O -3.137941 -1.817530 -0.153370
C -5.364417 -2.382445 -1.941601
C -7.838369 -0.722387 -0.717016
C -5.799257 0.649926 -0.971202
H -4.167653 0.327436 1.261612

H -4.290033 -1.098515 2.297577
H -6.650026 0.224056 1.781777
H -7.768743 -2.023828 1.729972
H -6.270448 -2.319621 2.597310
H -5.483098 -3.650089 0.764603
H -7.015328 -3.381808 -0.048679
H -8.409071 0.140494 -0.362607
H -7.914643 -0.738520 -1.806939
H -8.323566 -1.620451 -0.336115
H -6.356503 1.541903 -0.672109
H -4.750660 0.834877 -0.736082
H -5.884185 0.559714 -2.057099
H -6.329771 -2.637994 -2.383017
H -4.878618 -1.638896 -2.577211
H -4.741484 -3.279696 -1.946600
Na -1.117833 -0.815788 -0.084623
I 1.120110 -0.439721 -2.433706
Na 3.171270 0.408157 0.037419
I 1.010765 -0.557542 2.384560
I -2.071418 2.199134 -0.013516
Na 0.204806 2.633322 2.092240
I 2.492484 3.396612 0.092819
Na 3.164282 -2.750117 2.703826
I 4.600859 -2.462563 0.000745
Na 3.272584 -2.627864 -2.770508
Na 0.275943 2.720443 -2.024062

Na6I5.camphor+, V
E=-522.356176
C 4.926667 -1.576342 -0.431696
O 3.754964 -1.706725 -0.758809
C 5.475963 -0.625277 0.597246
C 6.095633 -2.351785 -1.005218
C 7.274202 -1.744697 -0.236124
C 7.179560 -2.291255 1.195790
C 5.916623 -1.580862 1.743698
C 6.835705 -0.267500 -0.067493
C 6.666714 0.488332 -1.383447
C 7.758411 0.544962 0.830932
H 6.316501 1.505853 -1.193719
H 5.952652 0.024893 -2.067494
H 7.625549 0.562272 -1.903367
H 7.376316 1.561488 0.951835
H 8.746980 0.620957 0.369488
H 7.888234 0.116786 1.824159
H 8.244533 -1.883489 -0.716709
H 7.082943 -3.378563 1.203180
H 8.064526 -2.042323 1.781675
H 5.115576 -2.272992 2.016518
H 6.132904 -0.981660 2.630799
H 5.962188 -3.425569 -0.848229
H 6.140693 -2.182748 -2.083721
H 4.211833 1.074754 0.154545
H 3.664507 0.089169 1.521285
H 5.045238 1.158366 1.712369
C 4.546634 0.485267 1.011367
Na 1.751731 -0.681503 -0.517103
I 1.542459 2.427848 -1.295461
Na 0.183405 2.653309 1.492543
I 0.518765 -0.301226 2.408478
Na -2.195853 -0.818659 1.122774
I -2.946845 2.362301 0.350637
Na -1.236078 1.731055 -2.332564
I -1.238272 -1.444719 -1.980102
Na -3.806672 -3.156847 -1.850469
Na -5.782317 1.210517 0.813027
I -5.191652 -1.793167 0.538660

Na6I5.camphor+, VI
E=-522.355592
C 6.290269 0.749470 1.742937
C 7.201933 1.223424 0.605570
C 6.955967 2.731947 0.458405
C 5.517239 2.782621 -0.114041
C 5.126640 1.291971 -0.327318
C 4.928717 0.795330 1.079327
C 6.501611 0.639073 -0.648000
C 7.108136 1.110471 -1.962753
C 6.437176 -0.886284 -0.673671
C 3.966047 1.057454 -1.257783
O 3.868950 0.475695 1.598885
Na 1.790021 -0.334774 1.294789
I -0.193339 1.938071 -0.539243
Na -0.348579 -0.615689 -2.444021
I -3.089048 -1.837537 -1.270086
Na -0.797765 -3.530579 0.517171
I -0.683192 -1.515285 2.879599
I 1.648779 -2.486406 -1.111301
Na -2.476239 0.316370 1.242767
I -4.834764 2.263716 0.281845
Na -2.445844 4.064843 -0.428718
Na -5.745434 -0.251716 -1.231769
H 6.492209 -0.272720 2.071650
H 6.310611 1.387270 2.630813
H 8.249566 0.939106 0.720666
H 7.673282 3.193089 -0.220736
H 7.042118 3.246561 1.417227
H 4.808723 3.289967 0.546332
H 5.475746 3.293846 -1.078399
H 8.134061 0.741342 -2.047268
H 6.546228 0.707064 -2.808649
H 7.136513 2.194575 -2.066690
H 7.445167 -1.306490 -0.723510
H 5.939230 -1.320070 0.195950
H 5.893535 -1.228922 -1.557327
H 4.209259 1.400715 -2.265989
H 3.702681 -0.001213 -1.309615
H 3.082207 1.608861 -0.927180

Na6I5.camphor+, VII
E=-522.354863
C 4.349110 2.672955 0.942340
C 4.723980 2.483419 -0.549587
C 3.547026 1.654237 -1.076177
C 3.198845 0.816693 0.129700
C 4.179168 1.146375 1.212210
C 5.519173 0.569549 0.662965
C 5.925228 1.532120 -0.479193
O 2.262838 0.021079 0.207058
C 3.810126 0.679968 2.597264
C 5.445952 3.316986 1.778311
C 3.055966 3.457865 1.153819
H 2.669371 2.241445 -1.354934
H 3.805200 1.015174 -1.927475
H 4.899984 3.408927 -1.100302
H 6.847442 2.063313 -0.244488
H 6.083181 1.011681 -1.425142
H 5.402041 -0.465091 0.327330
H 6.249127 0.558884 1.475218
H 5.649854 4.323201 1.402304
H 5.128341 3.416739 2.819088
H 6.384052 2.763459 1.767937
H 3.204445 4.501042 0.861706
H 2.212753 3.075599 0.574769
H 2.777070 3.447391 2.211456
H 4.498272 1.090966 3.338173
H 2.793999 0.972647 2.872112
H 3.893910 -0.408851 2.680572
Na -3.164257 -0.815994 1.528356
I -0.830688 -2.621076 2.444858
Na 2.021002 -1.989856 1.483077
I -2.433628 -1.438721 -1.613440
Na -0.888450 -4.011677 -0.477204
Na 0.692366 -0.383314 -1.658830
Na -3.150897 1.562932 -1.934049
I -3.940435 2.173025 1.086850
I 1.984930 -3.245339 -1.338523
I -0.186980 2.758505 -1.562169
Na -1.561466 4.059266 0.868081

Na6I5.camphor+, VIII
E=-522.330693
C 6.591440 -0.137920 0.027442
O 7.789808 -0.234920 0.156433
C 5.540127 -1.146292 0.474420
C 6.080463 -2.520434 0.770091
C 4.861432 -0.430154 1.668553
C 4.022590 0.702513 1.019027
C 4.380523 0.563601 -0.473901
C 5.833762 1.031107 -0.609001
C 4.497828 -0.970233 -0.658045
C 5.016216 -1.395147 -2.028140
C 3.181917 -1.698088 -0.403935
H 2.750396 -1.458165 0.570922
H 2.478334 -1.408861 -1.195269
H 3.337210 -2.780457 -0.460294
H 4.328673 -1.075072 -2.816601
H 5.096465 -2.483465 -2.081217
H 6.002758 -0.989882 -2.256511
H 6.844242 -2.463022 1.548041
H 6.551439 -2.959759 -0.111425
H 5.290695 -3.190464 1.119027
H 5.599920 -0.053663 2.379497
H 4.226381 -1.134328 2.212164
H 4.281832 1.690515 1.405917
H 2.970133 0.525667 1.253225
H 3.714275 1.049560 -1.199292
H 6.050058 1.964210 -0.083653
H 6.155877 1.152881 -1.645047
Na 1.449540 1.525744 -0.270136
I -0.225612 4.068269 -0.013798
Na -2.097478 2.541203 -1.904832
Na -1.692254 2.440143 2.131236
I -3.873020 0.849014 0.263838
I 0.140248 -0.238667 2.204149
I -0.299724 -0.125582 -2.441629
Na 0.397015 -2.435478 -0.204842
Na -3.186093 -1.523396 -1.893319
Na -2.818965 -1.616862 2.142605
I -2.357069 -3.774637 0.013518
